# Surveillance of RNase P, PMMoV, and CrAssphage in wastewater as indicators of human fecal concentration across urban sewer neighborhoods, Kentucky

**DOI:** 10.1093/femsmc/xtac003

**Published:** 2022-01-29

**Authors:** R. H. Holm, M. Nagarkar, R. A. Yeager, D. Talley, A. C. Chaney, J. P. Rai, A. Mukherjee, S. N. Rai, A. Bhatnagar, T. Smith

**Affiliations:** 1 Christina Lee Brown Envirome Institute, School of Medicine, University of Louisville, 302 E. Muhammad Ali Blvd., Louisville, KY 40202, USA; 2 Center for Environmental Solutions and Emergency Response, United States Environmental Protection Agency, Cincinnati, OH 45220, USA; 3 Department of Environmental and Occupational Health Sciences, School of Public Health and Information Sciences, University of Louisville, 485 E. Gray St., Louisville, KY 40202, USA; 4 Louisville/Jefferson County Metropolitan Sewer District, Morris Forman Water Quality Treatment Center, 4522 Algonquin Parkway, Louisville, KY 40211, USA; 5 Sanitation District No. 1 of Northern Kentucky, 1045 Eaton Dr., Ft. Wright, Kentucky 41017, USA; 6 Department of Bioinformatics and Biostatistics, School of Public Health and Information Sciences, University of Louisville, 505 S. Hancock St., Louisville, KY 40202, USA; 7 Brown Cancer Center, School of Medicine, University of Louisville, 505 S. Hancock St., Louisville, KY 40202, USA; 8 Center for Integrative Environmental Health Sciences, 500 S. Preston St., Suite 1319, Louisville, KY 40202, USA

**Keywords:** cross-assembly phage, fecal indicators, human ribonuclease P, pepper mild mottle virus, public health, sanitation

## Abstract

Wastewater surveillance has been widely used as a supplemental method to track the community infection levels of severe acute respiratory syndrome coronavirus 2. A gap exists in standardized reporting for fecal indicator concentrations, which can be used to calibrate the primary outcome concentrations from wastewater monitoring for use in epidemiological models. To address this, measurements of fecal indicator concentration among wastewater samples collected from sewers and treatment centers in four counties of Kentucky (N = 650) were examined. Results from the untransformed wastewater data over 4 months of sampling indicated that the fecal indicator concentration of human ribonuclease P (RNase P) ranged from 5.1 × 10^1^ to 1.15 × 10^6^ copies/ml, pepper mild mottle virus (PMMoV) ranged from 7.23 × 10^3^ to 3.53 × 10^7^ copies/ml, and cross-assembly phage (CrAssphage) ranged from 9.69 × 10^3^ to 1.85 × 10^8^ copies/ml. The results showed both regional and temporal variability. If fecal indicators are used as normalization factors, knowing the daily sewer system flow of the sample location may matter more than rainfall. RNase P, while it may be suitable as an internal amplification and sample adequacy control, has less utility than PMMoV and CrAssphage as a fecal indicator in wastewater samples when working at different sizes of catchment area. The choice of fecal indicator will impact the results of surveillance studies using this indicator to represent fecal load. Our results contribute broadly to an applicable standard normalization factor and assist in interpreting wastewater data in epidemiological modeling and monitoring.

## One sentence summary

Review of fecal indicators to foster a wider understanding of factors influencing wastewater results used in epidemiological modeling for public health surveillance.

## Introduction

Wastewater sampling for pharmaceuticals, personal care products, illicit drugs, and enteroviruses is well established; however, it lacks standardized reporting or the use of a positive control to calibrate results to account for differential fecal loading (Ort et al. 2010, 2014; Bisseux et al. 2020). Wastewater monitoring for severe acute respiratory syndrome coronavirus 2 (SARS-CoV-2) has rapidly expanded since it was first reported in early 2020 (Medema et al. 2020; Wu et al. 2020). Current guidelines for wastewater reporting are established for influent or effluent to the environment at treatment facilities for compliance, compliance assistance, civil and criminal investigations, and water quality studies (EPA 2017). Although there are no mandates on SARS-CoV-2 reporting, there are general guidelines for minimum meta-information necessary, including the use of an endogenous fecal indicator (McClary-Gutierrez et al. 2021). Wastewater-monitoring for SARS-CoV-2 is regarded as the assessment of a collection of pooled community stool samples for public health surveillance; however, the actual concentration of fecal indicators at all levels of sewer catchment is unknown despite its importance for the interpretation of results.

Normalizing target pathogen concentration measurements with a human fecal indicator concentration is one method to adjust for factors contributing to variability in the recovery and analysis of SARS-CoV-2. Commonly promoted fecal indicators include human ribonuclease P (RNase P; Peccia et al. 2020), pepper mild mottle virus (PMMoV; Bivins et al. 2020; Wu et al. 2020; D’Aoust et al. 2021; Jafferali et al. 2021), and cross-assembly phage (CrAssphage; Bivins et al. 2020; Green et al. 2020). RNase P is a human enzyme currently measured in nasal swab quantitative polymerase chain reaction (qPCR) testing to validate the adequate content of human samples (Food and Drug Administration 2020). PMMoV is a plant virus associated with peppers commonly found in the human diet and persists in the feces (Zhang et al. 2006; Hamza et al. 2011). CrAssphage is a bacteriophage infecting human gut commensal bacteria and is excreted in the feces (Dutilh et al. 2014; Stachler and Bibby 2014; Honap et al. 2020). These three are the ‘gold standard’ biomarkers associated with quantifying human signals; however, their utility as normalization factors for SARS-CoV-2 wastewater measurements depends on addressing several limitations. None of these potential biomarkers are enveloped viruses such as SARS-CoV-2. Therefore, the relative recovery of their signal may differ from that of SARS-CoV-2 and be impacted by different physicochemical characteristics within the wastewater. In addition, differences in capsid structures (helical vs. icosahedral) and genomes (RNA vs. DNA) influence decisions for downstream method (e.g. extraction and reverse transcriptase) selection. Furthermore, owing to spatial and temporal variations in the dilution of domestic wastewater, data do not exist to accurately estimate the amount or proportion of human feces contained in a set volume of a wastewater sample.

Although PMMoV (Rosario et al. 2009; Hamza et al. 2011, 2019; Kitajima et al. 2014; Kuroda et al. 2015; Schmitz et al. 2016; Gyawali et al. 2019; Malla et al. 2019; Tandukar, Sherchan and Haramoto 2020) and CrAssphage (García-Aljaro et al. 2017; Stachler et al. 2017; Ahmed et al. 2018; Farkas et al. 2019; Malla et al. 2019; Tandukar, Sherchan and Haramoto 2020) have been consistently detected in raw sewage, there are less data characterizing the relationship between the concentration of human fecal indicators and the wastewater signal of the target pathogen. In contrast, RNase P has not been commonly used in wastewater work as an indicator concentration in signal normalization. The influence of population size and household income has also not been well characterized when working at different sizes of sewer catchments for indicator concentrations.

The aim of this study was to assess RNase P, PMMoV, and CrAssphage as indicators of human fecal concentration across urban community sewersheds with different population sizes, income distributions, residence time, dilution, and daily flow. The results provide a wider understanding of how fecal indicator data are affected by sewer system factors and the populations they serve, which may influence their utility in wastewater surveillance and epidemiological modeling.

## Materials and methods

### Study site

**Figure 1.**
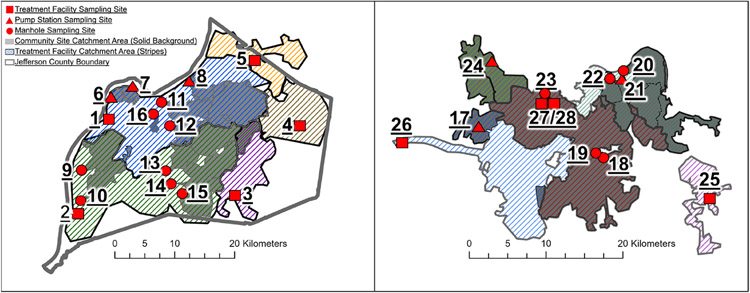
Location of wastewater sampling sites and corresponding catchment areas in Louisville/Jefferson County Metropolitan Sewer District (MSD) (left) and Sanitation District No. 1 (SD1) of Northern Kentucky (right). Numbered location identifiers are presented in Table 1. Solid colors indicate community sewersheds (manholes and pump stations) whereas diagonal lines with a white background are the larger treatment centers.

Two sewer systems within the commonwealth of Kentucky were sampled regularly during this study (Fig. 1): (i) the Louisville/Jefferson County Metropolitan Sewer District (MSD), and (ii) the Sanitation District No. 1 (SD1) of Northern Kentucky (NKY). In the city of Louisville/Jefferson County the sewer system is managed by the MSD and includes five water quality treatment centers (WQTC) serving approximately 770 000 residents. The MSD system contains active elements in operation for over a century and receives industrial wastewater ranging from 1% to 30%. Specifically, the five treatment centers include: Cedar Creek Water Quality Treatment Center (CCWQTC) 1%; Derek R. Guthrie Water Quality Treatment Center (DRGWQTC) 5%; Floyds Fork Water Quality Treatment Center (FFWQTP) 1%; Hite Creek Water Quality Treatment Center (HCWQTC) 30%; and Morris Forman Water Quality Treatment Center (MFWQTC) 10%. Within the system, the largest treatment center servicing the urban center, MFWQTC, combines rainwater runoff and domestic sewage in the same network pipes, and the remaining four regional WQTCs are separate sanitary sewer drainage. The sewer system managed by the SD1 spans Boone, Kenton, and Campbell counties and mostly is comprised of the suburbs of Cincinnati, Ohio, serving approximately 340 000 residents. Three WQTCs comprise SD1. Within the SD1 system, 6% is a combined sewer (31 km^2^), and the remainder is separate sanitary sewer drainage (471 km^2^).

During the study period, Kentucky was generally in a household-level stay-at-home order owing to the coronavirus disease 2019 (COVID-19); the Jefferson County school district (about 100 000 students) remained in virtual instruction.

### Sewage samples

**Figure 2.**
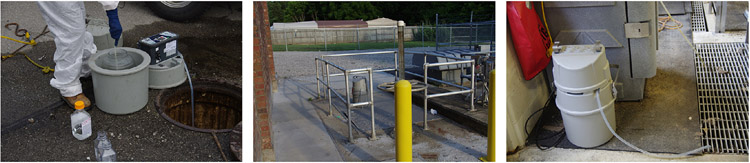
Sample collection types: street line manhole **(A)**, mechanical pump station **(B)**, and influent to water quality treatment center **(C)**.

Raw wastewater samples were collected from 16 sites to represent geographically distinct catchment areas in Louisville/Jefferson County, Kentucky (USA). There were three sample collection types (Fig. 2): (i) street line manholes, which are the closest to house-holds that contribute feces to a wastewater sample; (ii) mechanical pump stations, which represent a mid-point between manholes and WQTCs on secured sewer district property; and (iii) raw sewage flowing into the WQTCs before treatment. The selection protocol of the geographically resolved community wastewater sample sites was presented by Yeager et al. (2021). The field sample collection procedure is provided in Supplement A. The sewer district collected samples with a 24 hour time-weighted composite sampler, and a 30 ml volume was pulled every 15 minutes into a 4 l container. From this 4 l container, after stirring, a 125 ml aliquot was poured into a sample bottle. In the event of an equipment malfunction, such as a composite sampler battery problem or tubing clog, a grab sample was collected with a cup on a rope, which was applied to 15/566 samples. Samples were stored on ice during sampling and transportation to the University of Louisville laboratory. The composite samplers were stationary during the sample collection period. Samples were collected from 17 August to 17 December, 2020, one to four times per week. The measured daily total flow for WQTCs on the date of sample collection and a modeled flow rate for community site locations (manholes and pump stations) were provided by the MSD. The measured rainfall data for WQTCs during the 24 hour sample collection period were also provided by MSD; this was extrapolated to nested upstream contributing sites as appropriate.

Raw wastewater samples were additionally collected from 12 sites (manholes, pump stations, and WQTCs) serving Boone, Campbell, and Kenton Counties, in Northern Kentucky (USA). Samples in SD1 were collected using a 24 hour composite sampler. A volume of 125 ml was collected, stored on ice, and transported via overnight delivery to the University of Louisville laboratory. Samples from SD1 were collected from 3 September to 15 October, 2020, once per week.

### Fecal indicator detection and quantification

Full method details are provided by Fuqua et al. (2021). All samples were analyzed in the same laboratory at the University of Louisville. Samples were maintained on ice throughout the process, and 40 ml samples were processed within 12 hour of collection. Samples were clarified using a 70 μm cell strainer (Thermo Fisher Scientific, 22 363 548), concentrated overnight with polyethylene glycol incubation [5% PEG800 (Millipore-Sigma, 1546605); 0.2 M NaCl (VWR, 0241)] and pelleted by centrifugation at 16 000 × *g* for 30 minutes at 4°C. Pellets were resuspended in Trizol^™^ (Thermo Fisher Scientific, 15596018), and RNA was extracted using a Direct-zol^™^-96 MagBead RNA extraction kit (Zymo Research, R2102) per the manufacturer’s protocol. Eluted RNA was further purified from any contaminating substances using an RNeasy kit (Qiagen, 74104) and eluted from the column according to the manufacturer’s protocol. Thereafter, RNA quality was evaluated using a NanoDrop 1000 for concentration and purity. Samples resulting in RNA of sufficient quality (260/280 ratio > 1.9) and concentration (at least 10 ng/μl) were quantified with an Applied Biosystems QS3 RT PCR System for the copy number of RNase P, PMMoV, and CrAssphage. Less than 1% of the samples failed to meet these quality standards. Samples were analyzed in triplicates. Standard published primer/probe sets were used for all three targets (sequences are listed in Supplement Table B1; reverse transcription (RT)-qPCR operating conditions are summarized in Supplement Table B2). DNA plasmids containing the respective primer-probe regions were used to generate the standard curves. PCR inhibition was qualified in the method development by dilution of the RNA template. In 20+ samples across multiple weeks, the RNA template was diluted 1:3 before adding to the respective reaction mixture, and a corresponding Ct shift of 1 was anticipated. The average shift was 1.05. Data were reported on an unconcentrated sample basis (copies/ml of wastewater). In this study, we only reported on the RNase P, PMMoV, and CrAssphage values generated using this methodology.

### Data analysis

Samples with triplicate reactions amplified and above the detection limit (RNase P at 50 copies/ml, PMMoV at 143 copies/ml, and CrAssphage at 56 copies/ml) were considered. Averages of the triplicate results were used for data analysis. Population and income were based on the 2018 American Community Survey (ACS) (U.S. Census Bureau, 2019).

Data characteristics for MSD and SD1 include the following continuous variables: area, population, population density, and household income. The MSD sites additionally include: flow rate of sewer system site, the temperature of the wastewater sample at time of collection, and daily rainfall. In addition, the following categorical variables were assessed: sewer district (two levels; MSD or SD1), sample location type (three levels: manhole, pump station, or treatment center), and sample acquisition type (two levels: composite or grab for MSD only). We also aggregated the data from the 11 MSD manhole or pump station samples for comparison with data collected at the treatment center itself. We compared four groups which were within Louisville/Jefferson County (MSD): (i) MFWQTC, (ii) aggregate of samples leading to MFWQTC, (iii) Derek R. Guthrie Water Quality Treatment Center (DRGWQTC), and (iv) aggregate of samples leading to DRGWQTC. The outcome measures were RNase P, PMMoV, and CrAssphage. Population and income measures were presented in thousands. In addition, rainfall measurements were exponentiated, whereas days of no rainfall were replaced by a zero measurement because dividing by zero was not appropriate. Statistical analyses for RNase P, PMMoV, and CrAssphage were transformed using log base e, which improved normality. Outcome measures were generated by the different characteristics and were compared using a t-test (based on the generalized linear model owing to unbalanced ANOVA). Site variability of log_e_ for fecal indicators over the period of sample collection across catchment areas studied and across different site types (manholes, pump stations, and treatment centers) was compared using the Kruskal–Wallis test (Walker and Shostak 2010).

To apply the regression analyses, the class variables were converted into indicator variables. For example, manholes, pump stations, and treatment centers were binary indicator variables (0,1) derived from the sample location type. The data were partitioned into three subsets: only MSD sites (N = 566), MFWQTC (N = 67) and community sites leading to MFWQTC (n = 198), and DRGWQTC (n = 34) and samples leading to DRGWQTC (N = 165). Univariate and multivariate regression analyses were conducted on these three subsets for each outcome measure. Multivariable models included only significant characteristics at *α* = 0.05, based on univariable models. The results were considered statistically significant at *α* < 0.05. Data were analyzed using SAS 9.4 (Cary, N.C.).

### Ethics

The University of Louisville Institutional Review Board classified this project as Non-Human Subjects Research (reference #: 717950).

## Results and discussion

Over our study period, the untransformed wastewater data (i.e. copies/ml wastewater) of RNase P ranged from 5.1 × 10^1^ to 1.15 × 10^6^ copies/ml; PMMoV ranged from 7.23 × 10^3^ to 3.53 × 10^7^ copies/ml; and CrAssphage ranged from 9.69 × 10^3^ to 1.85 × 10^8^ copies/ml (Supplement Table C1).

When comparing the two areas of Kentucky sampled, MSD and SD1, the 28 sewershed areas (km^2^) were not significantly different from one another (*P* = 0.874; km^2^ for the 16 MSD sites compared to the 12 SD1 sites); however the log_e_ results were significantly different (*P* < 0.001) for RNase P, PMMoV, and CrAssphage (Supplement Table C2). A higher mean log_e_ concentration of RNase P was measured at MSD, whereas a higher mean log_e_ concentration for PMMoV and CrAssphage was measured at SD1. This indicates regional variability within the studied areas for the targets studied.

### Temporal trends

**Figure 3.**
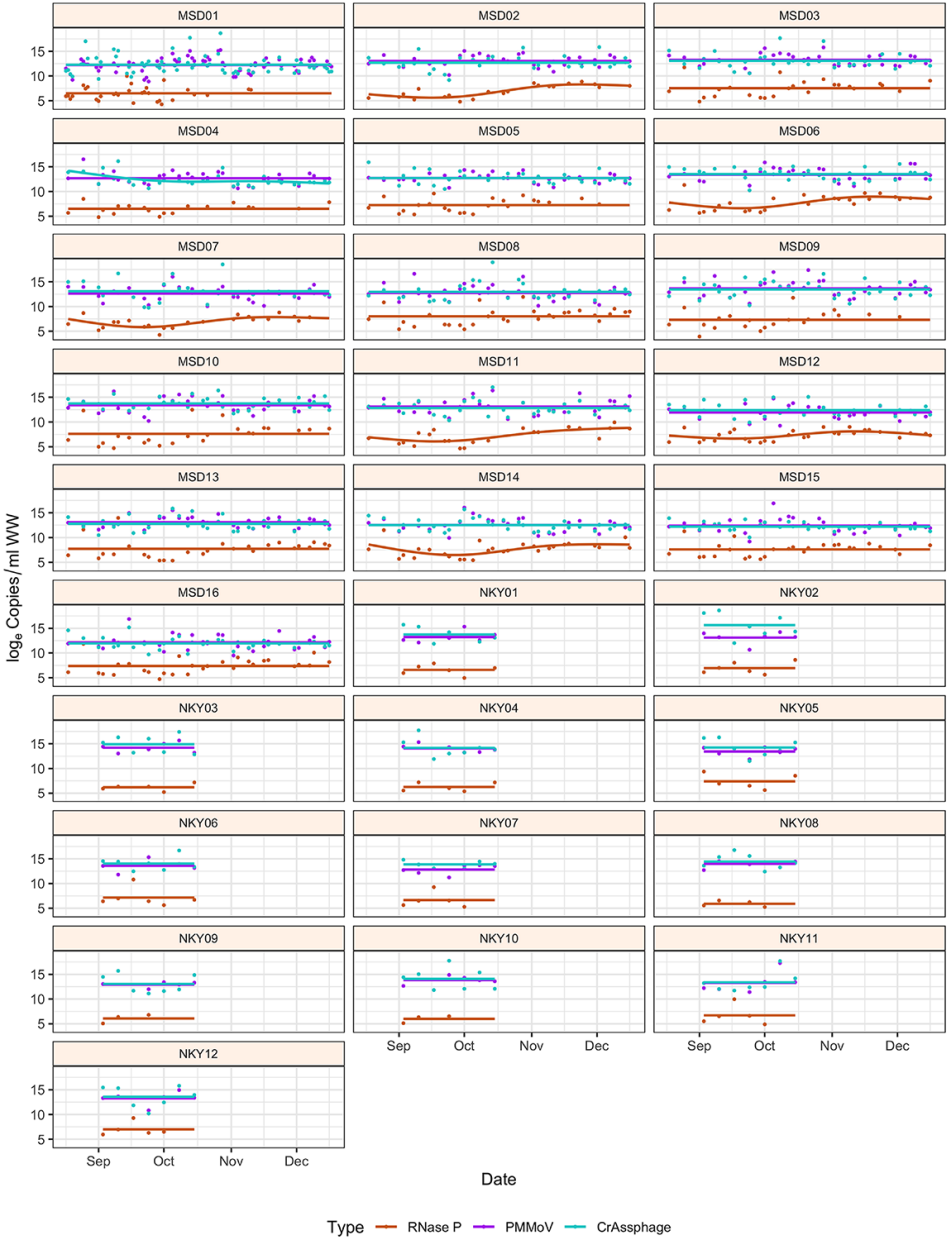
Temporal variability of log_e_ copies/ml for fecal indicators across Louisville/Jefferson County Metropolitan Sewer District (MSD) and Sanitation District No. 1 (SD1) of Northern Kentucky (NKY) sites from August to December 2020. The scatter plot represents the raw data, and the lines represent the best fit of fecal indicators as a function of time.

**Figure 4.**
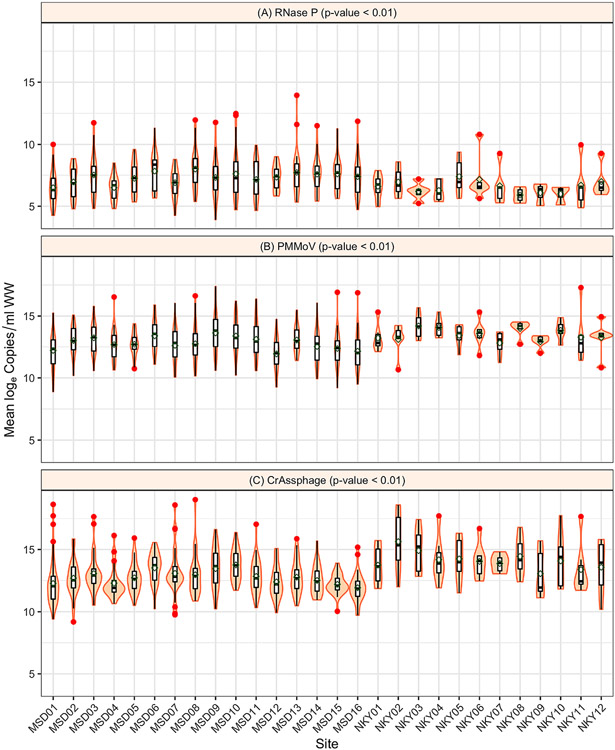
Site variability of log_e_ concentration for fecal indicators over the period of sample collection across catchment areas studied for Louisville/Jefferson County Metropolitan Sewer District (MSD) and Sanitation District No. 1 (SD1) of Northern Kentucky (NKY) sites for RNase P **(A)**, PMMoV **(B)**, and CrAssphage **(C)**. The shaded regions represent the distributions of log_e_ concentration, and the red dots represent the outliers. The *P*-values were based on the Kruskal–Wallis test.

In our study, fecal indicator concentration was measured for four months across 28 sewersheds of constant population sizes to determine the stability of fecal indicators over time (Fig. 3). A natural cubic spline with two change points was best fit to the data for the MSD sites, whereas for the SD1 sites, a linear model was fit as a function of time because of the smaller number of samples. An intercept-only model was selected when the spline or linear model was not significantly different. PMMoV and CrAssphage had more linear fits than RNase P; however, the variability in concentration was still across several orders of magnitude, suggesting that normalization attempts by RNase P may be less valid. In addition, among the 28 sites, the variability of log_e_ concentration results was significant (*P* < 0.01) for RNase P, PMMoV, and CrAssphage (Fig. 4). There was substantial heterogeneity in the variances across sites, although the variability in trends between MSD and SD1 sites might be due to sample size differences. In temporal trends, and consistent to our findings, Kitajima et al. (2014) and Hamza et al. (2019) also noted PMMoV concentration had no clear seasonal variation.

Stool generation location (at home, school, or employment) and when people defecate, is also a factor to be considered in wastewater sampling, as multiple defecations by the same person could contribute more fecal indicators to a wastewater sample and/or move across sewersheds during the same day. Global stool frequency ranges from 0.74 to 1.97 motions per 24 hour with a median of 1.10 defecations per 24 hour period; however, the frequency varies depending on the population primarily owing to fiber intake (Rose et al. 2015). Heaton et al. (1992) reported that in the UK, most adult defecations occurred in the morning (06:00–10:00), and few defecations were reported at 01:00–05:00. Our samples were collected as a 24 hour composite from the sewer network to remove any issues with people defecating at different times of the day. However, the impact of pandemic-associated stay-at-home orders on stool generation location over time is a poorly understood component of wastewater surveillance used in epidemiological modeling.

### Household sewer catchment size and income level

**Figure 5.**
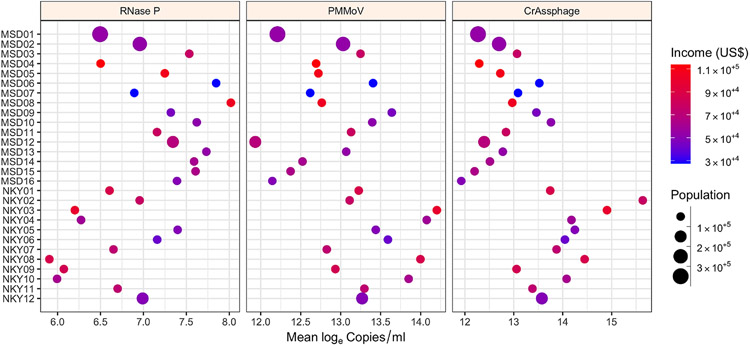
Log_e_ fecal indicators compared with household income (USD$) (mean within catchment areas of reported block group median yearly values) and total population size from 2018 U.S. Census Bureau American Community Survey for Louisville/Jefferson County Metropolitan Sewer District (MSD) and Sanitation District No. 1 (SD1) of Northern Kentucky (NKY) sites.

Each of the three fecal indicators was consistently present in the wastewater, regardless of catchment population size or income level (Fig. 5). However, larger population sizes were not necessarily associated with greater concentration of RNase P, PMMoV, or CrAssphage. The importance has been made clear for monitoring small populations where a few individuals excreting drugs into a sewershed can substantially affect wastewater relative loads, and even small sewersheds may have high drug compound concentrations (Ort et al. 2014); the same could be said for SARS-CoV-2 concentrations in wastewater where not everyone is excreting the virus. However, for fecal indicators, individuals within the same population might be expected to shed at approximately equal rates if their diet is the same, making the catchment basin scale less relevant when concentrations are being measured. An exception might be the impact by large influxes of other inputs such as stormwater or industrial wastewater. In our study, the sampling design intentionally maximized household units and limited industrial inputs. Our population findings contrast with those of Green et al. (2020), who found increasing CrAssphage concentrations in wastewater samples with increasing population catchment size over two weeks in Syracuse, New York, whereas other studies including a range of populations (García-Aljaro et al. 2017; Malla et al. 2019) have not well characterized the influence of catchment population size on human fecal indicator concentration in sewage for comparison to our work.

Geographic variations in diet and microbiomes have been suggested for PMMoV and CrAssphage variability (Bivins et al. 2020). We hypothesized income level could be an important factor associated with diet differences applicable at the city-scale contributing to an indicator concentration from feces. There were large differences (ranging from $27 000 to $114 000) in yearly mean median household income among the study locations. The two MSD sewersheds in West Louisville and East Downtown (MSD6 with an income of $28 000 and MSD7 with an income of $27 000) have an established inequity in food access compared to other areas of Louisville/Jefferson County (Mayor’s Healthy Hometown Movement 2010). However, our results showed income distributions were not necessarily associated with copy numbers/ml of RNase P, PMMoV, or CrAssphage. Rather, this suggests income distribution may not be a primary contributing factor for concentration variation observed in our intrastate-scale study, possibly owing to similar diet and body size of individuals.

### Grab and composite

We could only identify grab and composite samples for the MSD sample locations. The wastewater sample temperature in grab and composite samples at time of collection was significantly different (*P* < 0.001), with higher temperatures in grab samples (composite samples ranged from 33 to 69°F and grab samples ranged from 39 to 77°F; Supplement Table C3).

When grab and composite samples were further compared, log_e_ RNase P concentrations were different for the samples (*P* = 0.007), whereas for log_e_ PMMoV and CrAssphage concentrations, no differences were observed (*P* = 0.258 and *P* = 0.195, respectively). This could indicate in a study design with composite samples as the field protocol priority intent and in the limited case of grab samples collected in the morning hours, PMMoV and CrAssphage may be combined in the data set.

### Combined and non-combined systems

Our investigation would be considered to have been conducted in the dry season, the maximum 24 hour rainfall at a study site was 1.95 inches on 9 March 2020. There was no significant difference between areas with combined sewers (where high rainfall events may have caused dilution of fecal indicators from domestic sewage) or separated sewer systems for log_e_ RNase P copies/ml (*P* = 0.846) or log_e_ CrAssphage copies/ml (*P* = 0.051), but differences were seen for log_e_ PMMoV copies/ml (*P* < 0.001) (Supplement Table C4). Our results indicate that PMMoV varies with the addition of stormwater to the sewer system, whereas no effect was found for RNase P or CrAssphage. The explanation of PMMoV variability with stormwater input but not of the other fecal indicators needs further investigation, and possibly across a wider regional scale. Any change in composition in water could impact measurements of different types of viruses depending on how viruses interact with their physical or chemical environment. When the combined or separated system concentrations of the targets were further normalized by flow rate, differences were found for RNase P, PMMoV, and CrAssphage (*P* < 0.001); however, when alternatively normalized by site-specific 24 hour rainfall amounts, there was no difference in RNase P (*P* = 0.575), PMMoV (*P* = 0.122), or CrAssphage (*P* = 0.448). In addition, when the fecal indicators were normalized by a combined rainfall and flow normalization factor, the differences were significant (*P* < 0.001). These results indicate that flow correction for fecal indicators may matter more than a rainfall correction, or a combined rainfall and flow correction, when working with a complex-sewer-system scale including both combined and separated network pipes.

### Sample location and type

**Figure 6.**
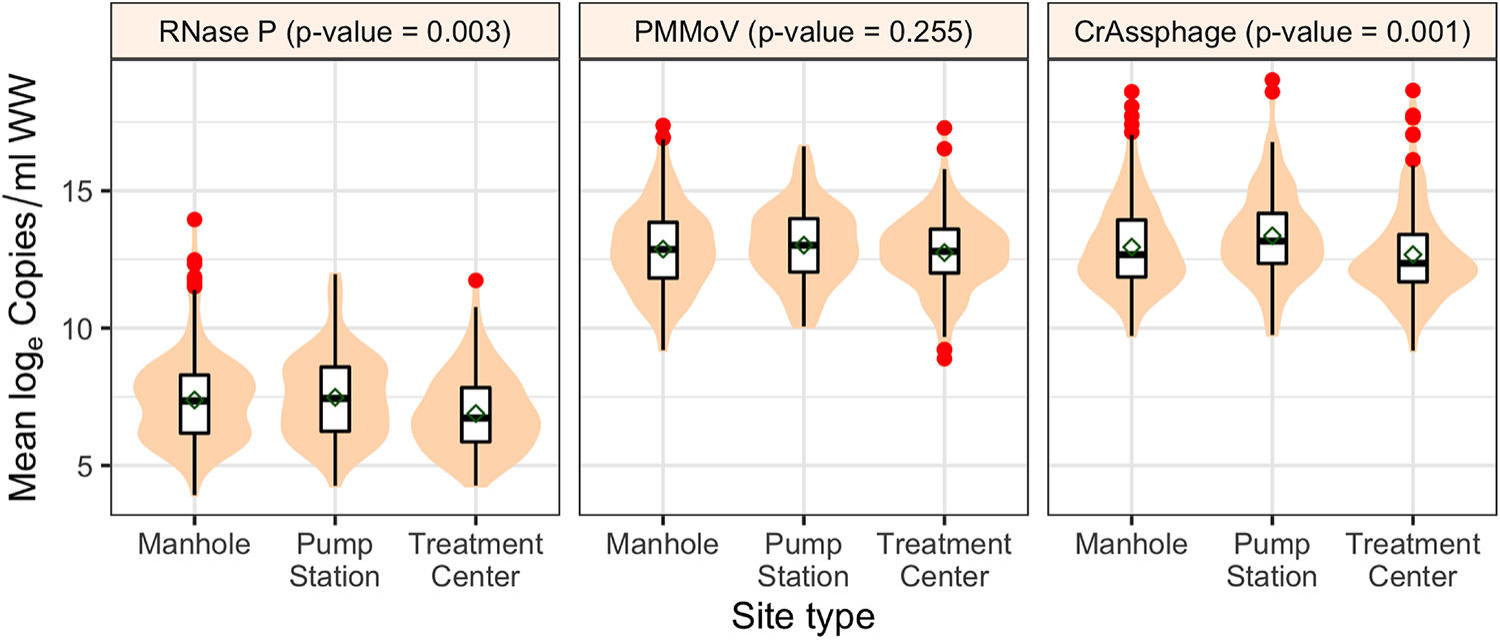
Comparison of log_e_ of fecal indicators across different sample location site types (manhole, pump station, and treatment center) for Louisville/Jefferson County Metropolitan Sewer District (MSD) and Sanitation District No. 1 (SD1) of Northern Kentucky (NKY) sites. The shaded regions represent the distributions of log_e_ concentration, and the red dots represent the outliers. The *P*-values were based on the Kruskal–Wallis test.

In a sewer system, manhole locations would be nearest to the stool generation sites, with additional travel time for samples collected at pump stations and even longer travel time to WQTCs. Among these sample collection types, there was limited variability in the sewer network infrastructure (Fig. 2). The log_e_ RNase P (*P* = 0.003) and log_e_ CrAssphage (*P* = 0.001) concentrations were different; however, there was no difference in log_e_ PMMoV concentration (*P* = 0.255) (Fig. 6; Supplement Table C5). This indicates PMMoV is more stable during sewer system travel, whereas RNase P and CrAssphage may have an interplay of extra processes when traveling from the manhole to the WQTC. These processes may need further study to isolate.

**Figure 7.**
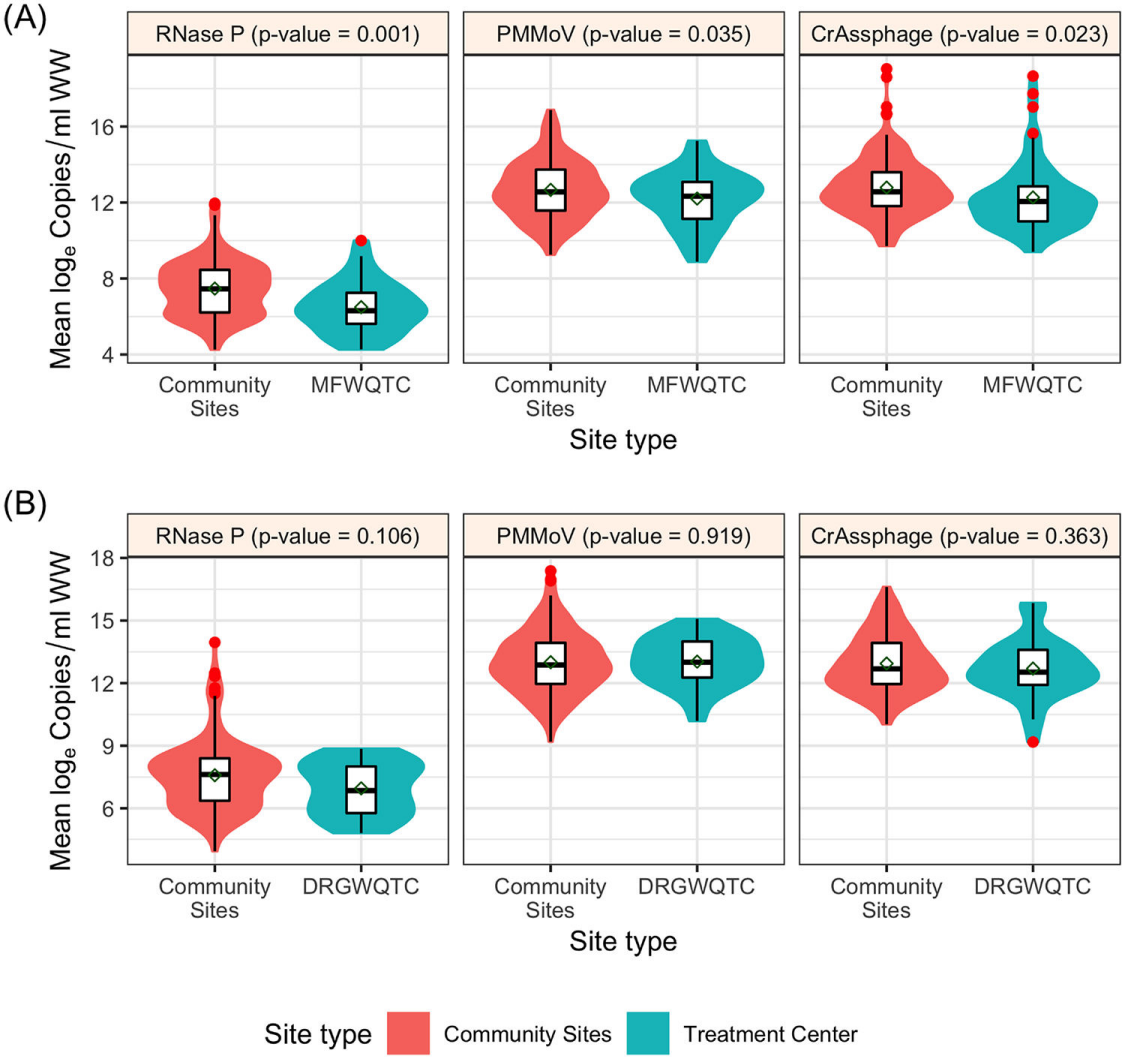
Log_e_ concentration of fecal indicators at aggregate sites (the treatment centers; shaded green) compared to that of nested upstream contributing sites (shaded orange). Morris Forman Water Quality Treatment Center (MFWQTC) (N = 6 contributing sewersheds) **(A)**; and Derek R. Guthrie Water Quality Treatment Center (DRGWQTC) (N = 5 contributing sewersheds) **(B)**. The shaded regions represent the distributions of log_e_ concentration, and the red dots represent the outliers. The *P*-values were based on the one-way ANOVA test.

Copy numbers/ml of fecal indicators at aggregate sites (the treatment centers) were compared to that of nested contributing sites to understand whether a WQTC can be assumed to represent the accumulation of its upstream sites (Fig. 7). Six upstream sewersheds that contribute to MFWQTC (industrial input ~10%; combined sewer system) were sampled. The log_e_ concentration between MFWQTC and contributing sites was significantly different for each RNase P (*P* = 0.001), PMMoV (*P* = 0.035), and CrAssphage (*P* = 0.023) (Supplement Table C6). In the second case, for DRGWQTC (industrial input ~5%; not a combined sewer system), the log_e_ concentration of RNase P, PMMoV, or CrAssphage was not significantly different between the WQTC and the five sampled contributing sites (*P* values of 0.106, 0.919, and 0.363, respectively) (Supplement Table C7). This warrants further study, as it suggests that at least in combined sewer system sewersheds, surveillance of fecal indicators at a finer geographic resolution may provide information that sampling at the WQTC alone could mask.

### Fecal indicators for use as a successful normalization factor

The regional and temporal variability found within the studied areas of Kentucky indicates that a constant fecal indicator denominator as a normalization factor is not appropriate, with variability seen in all three targets. PMMoV and CrAssphage concentrations appeared to be the most suitable fecal indicators for normalization. RNase P when tested as a normalization alternative to account for human cells has less utility when working at different geographic levels. Because samples were analyzed with consistent methods in the same laboratory for the study period, it is likely that the wide variation represents variability in natural fecal concentrations. However, future use of a recovery control may be useful for assessing consistency in lab processing losses.

Our ranges of PMMoV and CrAssphage concentrations were wide, with many outliers. Rosario et al. (2009) surveyed PMMoV across 11 states (USA), and the results were within a range of one order of magnitude. Furthermore, other global work most often shows a narrow range of magnitude (Hamza et al. 2011; Kitajima et al. 2014; Kuroda et al. 2015). Conversely, our PMMoV results ranged across four orders of magnitude. Our CrAssphage results also had a high range, across five orders of magnitude, but a wide range was similarly observed by Farkas et al. (2019). Comparisons between other data sets and across methodologies would require greater control to determine if the recoveries of the fecal indicators are different from those of SARS-CoV-2. If fecal indicator recoveries vary independently over time based on sample composition (such as pH and organic matter), that would make them poor normalization factors. The benefit of our study is the large sample size (N = 650) and constant field and laboratory methodology. Although none of the targets in this study period were homogeneous or stable, the results indicate that PMMoV and CrAssphage would likely remain more consistent over temporal and geographic levels of sewer catchment as successful normalization factors.

### Limitations

This study has several limitations, including limited data on regional and national-scale shedding rate variability of fecal indicators by individuals and defecation frequency and timing and, thus, the natural variability of input into the wastewater system. Reproducibility and sensitivity of laboratory methods to quantify fecal indicators in raw wastewater was not analyzed. The impact of influxes of stormwater and/or industrial wastewater for manholes and pump stations was not able to be observed.

## Conclusion

Investigating factors influencing the levels of fecal markers is critically important to wastewater-based epidemiology to appropriately characterize the denominator of chemicals and pathogens of interest. This is the first variable catchment-scale study of simultaneous RNase P, PMMoV, and CrAssphage wastewater concentrations. The results of this study of 650 samples in a four-month window indicate wide variations in target concentrations across population sizes, income distributions, residence time, dilution, and daily flow. RNase P, while it may be suitable as an internal amplification and sample adequacy control, has less utility than PMMoV and CrAssphage as a fecal indicator of wastewater samples when working at different geographic levels. Further studies are needed to determine the adjustment to other environmental, contextual, and population metrics and the accuracy of estimates after adjustments are made; at geographic scales across other regional and national cities; and for the application to SARS-CoV-2 surveillance. The choice of the fecal indicator will impact the results of surveillance studies using this indicator to represent fecal load. Our results contribute broadly to an applicable standard normalization factor and assist in the interpretation of wastewater data in epidemiological modeling and monitoring.

## Supplementary Material

Supplementary Data

## Figures and Tables

**Table 1.** Sampling site characteristics in Louisville/Jefferson County Metropolitan Sewer District (MSD) and Sanitation District No. 1 (SD1) of Northern Kentucky (NKY).

Site name	Map ID	Site characteristics						Flow rate (MGD)	
		Sewer district	Site type	Income (USD$)^a^	Population^a^	Area (km^2^)	Combined sewer	Mean ± SE	Median (min–max)
MSD01Morris Forman Water Quality Treatment Center (MFWQTC)	1	MSD	Treatment center	54 138	349 850	280	Yes	68.68 ± 2.98	62.99 (1.24–169.35)
MSD02Derek R. Guthrie Water Quality Treatment Center (DRGWQTC)	2		Treatment center	53 577	295 910	332	No	45.39 ± 3.16	40.74 (30.55–115.61)
MSD03Cedar Creek Water Quality Treatment Center (CCWQTC)	3		Treatment center	76 606	55 928	80	No	5.22 ± 0.34	4.86 (3.43–13.01)
MSD04Floyds Fork Water Quality Treatment Center (FFWQTC)	4		Treatment center	113 699	32 460	88	No	2.92 ± 0.25	2.64 (2.03–10.66)
MSD05Hite Creek Water Quality Treatment Center (HCWQTC)	5		Treatment center	106 769	31 269	67	No	4.02 ± 0.18	4.08 (0.02–6.51)
MSD06Shawnee Park PS	6		Pump station	27 695	10 739	5	Yes	39.78^c^	
MSD0734th Street PS	7		Pump station	27 446	7820	5	Yes	0.25^c^	
MSD08Muddy Forks PS	8		Pump station	103 304	11 203	12	Yes	0.98^c^	
MSD09MH32985	9		Manhole	45 895	35 956	28	No	0.01^c^	
MSD10MH09837	10		Manhole	51 656	25 073	21	No	1.06^c^	
MSD11MH08915A CSO140	11		Manhole	77 842	99 061	80	Yes	0.06^c^	
MSD12MH50495 CSO108	12		Manhole	68 259	139 251	112	Yes	5.04^c^	
MSD13MH23290	13		Manhole	53 542	73 666	55	No	23.06^c^	
MSD14MH57769	14		Manhole	61 837	46 659	37	No	7.12^c^	
MSD15MH57350	15		Manhole	63 642	22 437	23	No	4.17^c^	
MSD16MH71910 CSO146	16		Manhole	49 031	8071	3	Yes	2.00^c^	
NKY01	17	SD1	Pump station	86 250	15 073	16	No	^d^	
NKY02	18		Manhole	76771	33 988	73	No	^d^	
NKY03	19		Manhole	98 434	10 426	9	No	^d^	
NKY04	20		Manhole	59 011	39 194	39	Yes	^d^	
NKY05	21		Pump station	48 708	31 142	27	Yes	^d^	
NKY06	22		Manhole	41 750	15 147	8	Yes	^d^	
NKY07	23		Manhole	71 896	90 209	184	No	^d^	
NKY08	24		Pump station	85 515	9624	37	No	^d^	
NKY09	25		Treatment center	83 514	4899	31	No	^d^	
NKY10	26		Treatment center	61 811	95 565	127	No	^d^	
NKY11^b^	27		Treatment center	71 896	90 209	184	No	^d^	
NKY12^b^	28		Treatment center	48 740	113 705	124	Yes	^d^	

a Based on 2018 U.S Census Bureau American Community Survey (ACS). Income is mean median household.

b This location has two sampling locations with two distinct influents, the two sources were sampled separately.

c Modeled flow rate, based on dry season.

d Flow rate not available.

## Abbreviations

CCWQTC: Cedar Creek Water Quality Treatment Center
CrAssphage: cross-assembly phage
DRGWQTC: Derek R. Guthrie Water Quality Treatment Center
HCWQTP: Hite Creek Water Quality Treatment Center
FFWQTP: Floyds Fork Water Quality Treatment Center
RNase P: human ribonuclease P
MSD: Louisville/Jefferson County Metropolitan Sewer District
MFWQTC: Morris Forman Water Quality Treatment Center
NKY: Northern Kentucky sample site
PMMoV: pepper mild mottle virus
SD1: Sanitation District No. 1 of Northern Kentucky
SARS-CoV-2: severe acute respiratory syndrome coronavirus 2
