# Supplementary material for: Surveillance of RNase P, PMMoV, and CrAssphage in wastewater as indicators of human fecal concentration across urban sewer neighborhoods, Kentucky
Source: FEMS Microbes. Author manuscript; Available in PMC 2023 May 24. (PMC10117713; doi:10.1093/femsmc/xtac003)
Supplement: Supplementary Data [file NIHMS1895999-supplement-Supplementary_Data.docx]

**Supplementary Material**

**Surveillance of RNase P, PMMoV, and CrAssphage in wastewater as indicators of human fecal concentration across urban sewer neighborhoods, Kentucky**

R. H. Holm^a*^, M. Nagarkar^b^, R. A. Yeager^a,c^, D. Talley^d^, A. C. Chaney^e^, J. P. Rai^f^, A. Mukherjee^f^, S. N. Rai ^a,f,g,h^, A. Bhatnagar^a^ and T. Smith^a^

^a^Christina Lee Brown Envirome Institute, School of Medicine, University of Louisville, 302 E. Muhammad Ali Blvd., Louisville, KY 40202, United States

^b^Center for Environmental Solutions and Emergency Response, United States Environmental Protection Agency, Cincinnati, OH 45220, United States

^c^Department of Environmental and Occupational Health Sciences, School of Public Health and Information Sciences, University of Louisville, 485 E. Gray St., Louisville, KY 40202, United States

^d^Louisville/Jefferson County Metropolitan Sewer District, Morris Forman Water Quality Treatment Center, 4522 Algonquin Parkway, Louisville, KY 40211, United States

^e^Sanitation District No. 1 of Northern Kentucky, 1045 Eaton Dr., Ft. Wright, KY 41017, United States

^f^Department of Bioinformatics and Biostatistics, School of Public Health and Information Sciences, University of Louisville, 505 S. Hancock St., Louisville, KY 40202, United States

^g^Brown Cancer Center, School of Medicine, University of Louisville, 505 S. Hancock St., Louisville, KY 40202, United States

^h^Center for Integrative Environmental Health Sciences, 500 S. Preston St., Suite 1319, Louisville, KY 40202, United States

*Corresponding author: Rochelle H. Holm (rochelle.holm@louisville.edu)

**Supplement A**

***Standard Operating Procedure (SOP) for wastewater sampling, transport, and storage for SARS-CoV-2 RNA assays***

**Purpose**

The purpose of this procedure is to collect samples from 24 h composite samplers installed at wastewater treatment facilities, pump stations, or manholes, and the subsequent transport and storage of samples.

Always follow field safety measures outlined in the ‘*Health and Safety Plan for Research on Wastewater-Based Epidemiology in Louisville*:’

- Wash hands before and after field activities.
- Use hand sanitizer after the collection of each sample.
- Wear Personal Protective Equipment.
- Do not eat or drink during field activities.

**Chain of custody**

Collected samples are in the custody of the sampler or sample custodian until the samples are relinquished to another party. The chain of custody should include the following real-time documentation of all activities:

- Record the sample identification.
- Record date and time of sample collection.
- Record any quality-control samples collected (for example, field blank or field duplicate).
- Record any other relevant information (for example, sample was turbid).
- Record any deviations from this protocol.

**Ethics**

It shall be the policy of the project to conduct all sampling, transport, and storage with integrity and in an ethical manner. It is a basic and expected responsibility of each staff member to hold the highest ethical standard of professional conduct in the performance of all duties.

**Sample collection equipment**

Ensure that the equipment is accounted for and works properly before arriving in the field to collect samples.

1. Sample containers: two sterile 125 ml polyethylene terephthalate (PET) bottles per site
2. Permanent markers
3. 70% ethanol in a 700 ml spray bottle (210 ml water + 200 proof ethanol up to the 700 ml mark on the bottle)
4. Paper towels
5. Ice
6. Battery-operated hand pump (1 per site)
7. Backup batteries for the hand pumps
8. Two backup hand pumps
9. Backup thermometer
10. Backup funnel
11. 18-inch plastic stir rod
12. Temperature probe
13. 10% bleach solution (400 ml per site)
14. 1 l glass bottle for the bleach solution
15. Plastic cooler box
16. Large plastic bag
17. Personnel protective equipment: hard hat, gloves, surgical face mask, nitrile gloves (minimum)
18. Camera
19. Logbook

**Sample preparation procedure**

1.
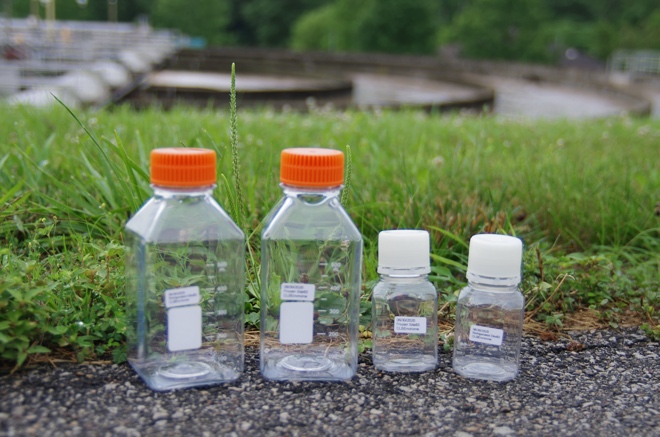
On the day before sampling, make labels for two 125 ml PET bottles per site. Number the samples in ascending order. Each site will receive a unique sample number, the collection date on the label, and the project name. Label one bottle per sample site “refrigerated” and one “frozen.” Affix the labels to each bottle.

When printing is complete, the label will look like this:

06/30/20

Frozen Site 84

CLBEnvironme

06/30/20

Refrigerated Site 84

CLBEnvironme

**Sample collection procedure**

1. On the day of sampling, prepare a plastic cooler box. Fill the bottom 1/3 of the cooler with wet and loose ice.
2. Travel to the sample site. ­­
3. Wear personnel protective equipment (gloves, mask, protective eyewear, firmly built and closed-toed footwear, long pants, and a hard hat). Use new gloves at every sample site.
4. Before sampling, prepare the equipment for easy access. Remove the caps from the two sample bottles, and place the caps on a paper towel nearby. It is helpful to have a large, low-sided plastic bin to stage sampling supplies and equipment.


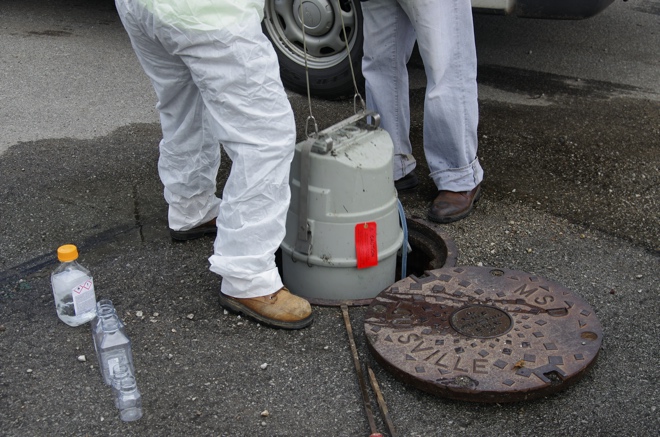


1. Retrieve and open the container holding the 24 h composite and iced wastewater sampler.


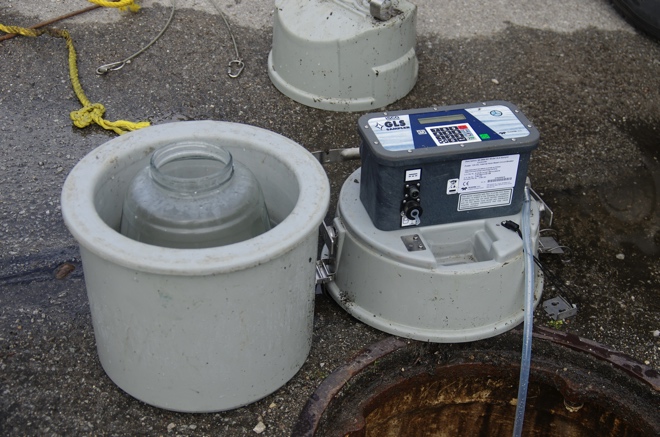


1. Stir the contents of the composite sampling container with an 18-inch plastic rod by hand for 10 s.


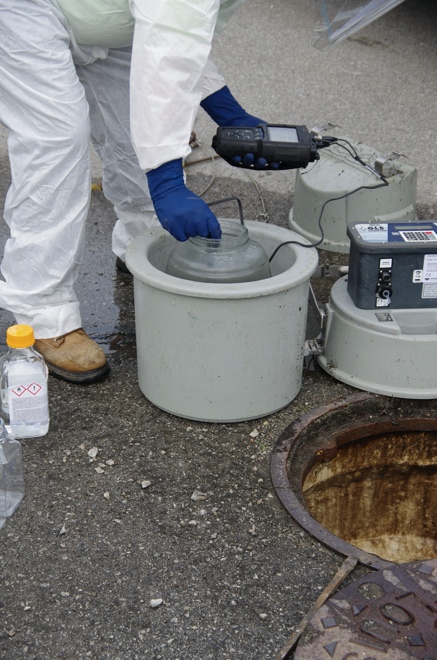


1. Place the temperature probe into the composite sampling container. Record the temperature in the chain-of-custody form.
2.
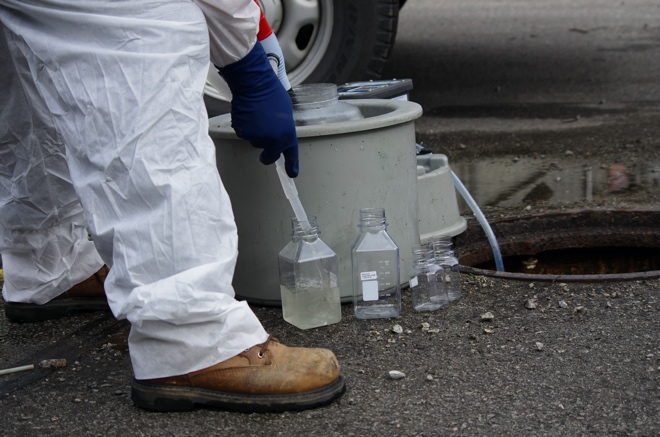
Using a battery-operated handpump, transfer sample from the composite sampler to the sample bottle. Fill each up to the shoulder of the sample bottle.
3. Record sample ID, sampling location, and sample collection time and date on the chain-of-custody form.
4.
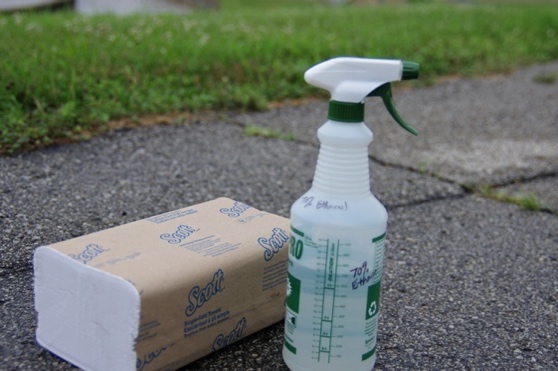
Close each sample bottle securely. Spray 70% ethanol onto paper towels, and wipe the outside of each sample bottle.
5. Place the filled bottles upright in the plastic cooler box with ice, and keep the lid closed.
6. Fill the 1 l glass bottle with 400 ml of 10% bleach solution. Place the handpump tubing into the glass jar and flush with approximately 300 ml of bleach solution through a battery-operated hand pump. Pour approximately 100 ml 10% bleach solution over the exterior of the pump.


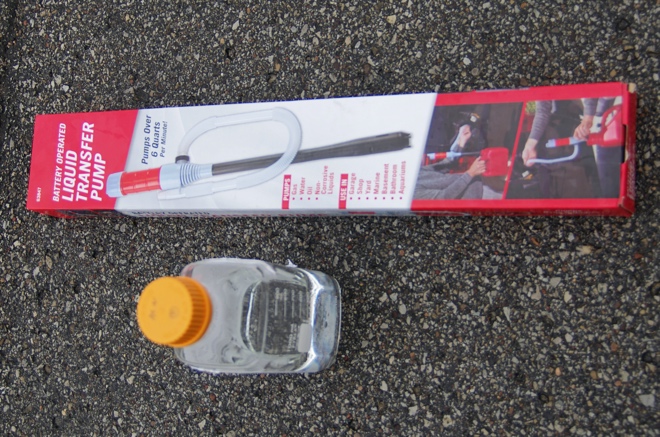


1. Place the handpump into the large plastic bag to return to the laboratory for cleaning.
2. Discard extra liquid contents of composite sample back into wastewater system.
3. Repeat steps 2 to 15 as needed to complete planned site visits.
4. When all sample sites have been visited, fill and sign the chain-of-custody form, including the date and time. Immediately transport samples to the laboratory.


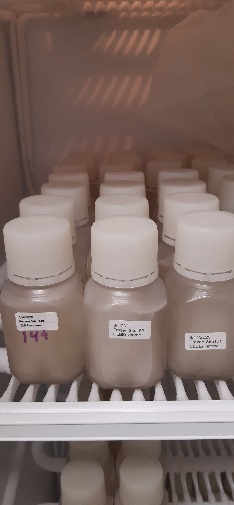


1. Start sample processing as quickly as possible. Line samples in a numeric order on a lab bench. Verify that the lids are tightly closed, and wipe each sample bottle with 70% ethanol sprayed onto paper towels. Deliver bottles marked ‘Refrigerated’ to the laboratory. Place bottles marked ‘Frozen’ in a -80°C freezer.
2. Clean with 70% ethanol sprayed onto paper towels or soap and water, and dry and store sample collection equipment (including the plastic cooler box, 70% ethanol spray bottle, eye protection goggles, and hard hat) for subsequent use. Dispose of nitrile gloves and surgical face masks in municipal trash.

# Attachment 1: Field check list for sampling supplies

The following should be loaded into the transport vehicle prior to departure:

- - A copy of this Standard Operating Procedure
  - Communication device (such as cell phone or 2-way radio)
  - A trash bag to hold used gloves and soiled paper towels
- Sample containers: two prelabeled 125 ml PET bottles per site
- Permanent marker
- 70% ethanol in a 700 ml spray bottle (210 ml water + 200 proof ethanol up to the 700 ml mark on bottle)
- Paper towels
- Ice
- Battery-operated hand pump (1 per site)
- Backup batteries for the hand pumps
- Two backup handpumps
- Backup thermometer
- Backup funnel
- 10% bleach solution (400 ml per site). Approximately 4 l total.
- 1 l glass bottle for the bleach solution
- Cooler box
- Large plastic bag
- 18-inch plastic stir rod
- Temperature probe
- Chain-of-custody form
- Camera
- Logbook

# Attachment 2: Field check list for personnel protective equipment

The following should be loaded into the transport vehicle prior to departure:

- - Basic first aid kit
  - Drinking water for the field team
  - Nitrile (or similar) gloves (no latex and recommend powder-free gloves)
  - Disposable surgical face masks
  - Protective eyewear and/or face shield
  - Firmly built and closed-toed footwear, long pants, and a hard hat
  - Soap available for handwashing at the end of each day
  - Hand sanitizer

**Supplement B**

***Table B1. Primer and probe sequences used for RT-qPCR in this study***

| **Target** | **Primer Name** | **Sequence (5’→3’)** | **Probes** |
| --- | --- | --- | --- |
| N1 | 2019-nCoV_N1-F | 5’-GACCCCAAAATCAGCGAAAT-3’ | None |
|  | 2019-nCoV_N1-R | 5’-TCTGGTTACTGCCAGTTGAATCTG-3’ | None |
|  | 2019-nCoV_N1-P | 5’-**FAM**-ACCCCGCATTACGTTTGGTGGACC-**QSY**-3’ | FAM, BHQ-1 |
| RNase | RNase P-F | 5’-AGATTTGGACCTGCGAGCG-3’ | None |
|  | RNase P-R | 5’-GAGCGGCTGTCTCCACAAGT-3’ | None |
|  | RNase P-P | 5’-**JUN**-TTCTGACCTGAAGGCTCTGCGCG-**QSY**-3’ | JUN, BHQ-1 |
| ORF | CoV_ORF1ab-F | 5’-GTCGTAGTGGTGAGACACTTG-3’ | None |
|  | CoV_ORF1ab-R | 5’-GGCCACCAGCTCCTTTATTA-3’ | None |
|  | CoV_ORF1ab-P | 5’-**FAM**-ATACCAGTGGCTTACCGCAAGGTT-**QSY**-3’ | FAM, BHQ-1 |
| PMMoV | PMMoV-F | 5’-GAGTGGTTTGACCTTAACGTTTGA-3’ | None |
|  | PMMoV-R | 5’-TTGTCGGTTGCAATGCAAGT-3’ | None |
|  | PMMoV-P | 5’-**VIC**-CCTACCGAAGCAAATG-**QSY**-3’ | VIC, BHQ-1 |
| CrAssphage | CrAssphage-F | 5’-CAGAAGTACAAACTCCTAAAAAACGTAGAG-3’ | None |
|  | CrAssphage-R | 5’-GATGACCAATAAACAAGCCATTAGC-3’ | None |
|  | CrAssphage-P | 5’-**JUN**-AATAACGATTTACGTGATGTAAC-**QSY**-3’ | JUN, BHQ-1 |

***Table B2. RT-qPCR operating conditions in this study***

| **Step** | **Stage** | **Cycles** | **Temperature (°C)** | **Time (Fast)** |
| --- | --- | --- | --- | --- |
| Uracil N-glycosylase (UNG) incubation | 1 | 1 | 25 | 2 mins |
| Reverse Transcription | 2 | 1 | 53 | 10 mins |
| Polymerase Activation | 3 | 1 | 95 | 2 mins |
| Amplification | 4 | 45 | 95 | 5 s |
|  |  |  | 60 | 30 s |

**Supplement C**

**Data Analysis**

***Table C1. Summary statistics of fecal indicators in raw wastewater by sampling site in Louisville/Jefferson County Metropolitan Sewer District (MSD) and Sanitation District No. 1 (SD1) of Northern Kentucky (NKY)***

***Table C2: Comparison of Louisville/Jefferson County Metropolitan Sewer District (MSD) and***

***Sanitation District No. 1 (SD1) of Northern Kentucky***

| **Variables** | **Total (N=650)** | **MSD (N=566)** | **SD1 (N=84)** | **P Value** |
| --- | --- | --- | --- | --- |
| **Area** |  |  |  | *0.874* |
| N | 28 | 16 | 12 |  |
| Mean ± SE | 74.47 ± 15.69 | 76.69 ± 23.99 | 71.51 ± 19.05 |  |
| Median (min - max) | 37.86 (2.96 - 331.83) | 45.91 (2.96 - 331.83) | 37.86 (7.94 - 183.81) |  |
| **Population** |  |  |  | *<.001* |
| N | 650 | 566 | 84 |  |
| Mean ± SE | 88.08 ± 4.33 | 94.36 ± 4.88 | 45.77 ± 4.20 |  |
| Median (min - max) | 35.96 (4.90 - 349.85) | 35.96 (7.82 - 349.85) | 32.57 (4.90 - 113.71) |  |
| **Income** |  |  |  | *0.054* |
| N | 650 | 566 | 84 |  |
| Mean ± SE | 64.90 ± 0.92 | 64.21 ± 1.02 | 69.52 ± 1.86 |  |
| Median (min - max) | 59.01 (27.45 - 113.70) | 54.14 (27.45 - 113.70) | 71.90 (41.75 - 98.43) |  |
| **Log_e_ RNase P (copies/ml)** |  |  |  | *<.001* |
| N | 463 | 405 | 58 |  |
| Mean ± SE | 7.26 ± 0.07 | 7.35 ± 0.08 | 6.64 ± 0.17 |  |
| Median (min - max) | 7.16 (3.92 - 13.95) | 7.37 (3.92 - 13.95) | 6.46 (4.89 - 10.81) |  |
| **Log_e_ PMMoV (copies/ml)** |  |  |  | *<.001* |
| N | 604 | 534 | 70 |  |
| Mean ± SE | 12.85 ± 0.06 | 12.77 ± 0.06 | 13.49 ± 0.14 |  |
| Median (min - max) | 12.87 (8.89 - 17.38) | 12.70 (8.89 - 17.38) | 13.48 (10.66 - 17.29) |  |
| **Log_e_ CrAssphage (copies/ml)** |  |  |  | *<.001* |
| N | 631 | 549 | 82 |  |
| Mean ± SE | 12.92 ± 0.06 | 12.75 ± 0.06 | 14.10 ± 0.20 |  |
| Median (min - max) | 12.60 (9.18 - 19.03) | 12.48 (9.18 - 19.03) | 13.98 (10.19 - 18.61) |  |

***Table C3: Comparison of the characteristics for Louisville/Jefferson County Metropolitan Sewer District (MSD) sites by grab or composite sample acquisition type***

| **Variables** | **Total (N=566)** | **Composite (N=551)** | **Grab (N=15)** | **P Value** |
| --- | --- | --- | --- | --- |
| **Area** |  |  |  | *0.101* |
| N | 566 | 551 | 15 |  |
| Mean ± SE | 89.35 ± 4.31 | 90.51 ± 4.41 | 46.52 ± 8.27 |  |
| Median (min - max) | 54.85 (2.96 - 331.83) | 54.85 (2.96 - 331.83) | 54.85 (2.96 - 111.56) |  |
| **Population** |  |  |  | *0.176* |
| N | 566 | 551 | 15 |  |
| Mean ± SE | 94.36 ± 4.88 | 95.45 ± 5.00 | 54.34 ± 9.65 |  |
| Median (min - max) | 35.96 (7.82 - 349.85) | 35.96 (7.82 - 349.85) | 55.93 (8.07 - 139.25) |  |
| **Population density** |  |  |  | *0.053* |
| N | 566 | 551 | 15 |  |
| Mean ± SE | 1222.25 ± 23.06 | 1214.90 ± 23.14 | 1492.26 ± 178.16 |  |
| Median (min - max) | 1248.16 (369.54 - 2730.46) | 1248.16 (369.54 - 2730.46) | 1343.04 (466.09 - 2730.46) |  |
| **Income** |  |  |  | *0.417* |
| N | 566 | 551 | 15 |  |
| Mean ± SE | 64.21 ± 1.02 | 64.35 ± 1.04 | 59.18 ± 4.30 |  |
| Median (min - max) | 54.14 (27.45 - 113.70) | 54.14 (27.45 - 113.70) | 53.54 (45.90 - 106.77) |  |
| **Flow (MGD)** |  |  |  | *0.264* |
| N | 561 | 546 | 15 |  |
| Mean ± SE | 16.20 ± 1.06 | 16.39 ± 1.08 | 9.07 ± 2.67 |  |
| Median (min - max) | 4.17 (0.01 - 169.35) | 4.17 (0.01 - 169.35) | 4.19 (0.01 - 23.06) |  |
| **Temperature (°F) at time of collection** |  |  |  | *<.001* |
| N | 497 | 484 | 13 |  |
| Mean ± SE | 38.41 ± 0.19 | 38.13 ± 0.15 | 48.77 ± 3.28 |  |
| Median (min - max) | 38.00 (33.00 - 77.00) | 38.00 (33.00 - 69.00) | 43.00 (39.00 - 77.00) |  |
| **Log_e_ RNase P (copies/ml)** |  |  |  | *0.007* |
| N | 405 | 392 | 13 |  |
| Mean ± SE | 7.35 ± 0.08 | 7.31 ± 0.08 | 8.47 ± 0.39 |  |
| Median (min - max) | 7.37 (3.92 - 13.95) | 7.31 (3.92 - 13.95) | 8.14 (6.90 - 12.48) |  |
| **Log_e_ PMMoV (copies/ml)** |  |  |  | *0.258* |
| N | 534 | 519 | 15 |  |
| Mean ± SE | 12.77 ± 0.06 | 12.76 ± 0.06 | 13.18 ± 0.29 |  |
| Median (min - max) | 12.70 (8.89 - 17.38) | 12.69 (8.89 - 17.38) | 12.72 (11.70 - 15.28) |  |
| **Log_e_ CrAssphage (copies/ml)** |  |  |  | *0.195* |
| N | 549 | 534 | 15 |  |
| Mean ± SE | 12.75 ± 0.06 | 12.73 ± 0.06 | 13.24 ± 0.36 |  |
| Median (min - max) | 12.48 (9.18 - 19.03) | 12.46 (9.18 - 19.03) | 12.75 (11.58 - 15.76) |  |
| **Log_e_ RNase P (copies/ml)/Flow (MGD)** |  |  |  | *0.415* |
| N | 403 | 390 | 13 |  |
| Mean ± SE | 65.74 ± 11.95 | 63.97 ± 11.85 | 119.12 ± 107.07 |  |
| Median (min - max) | 1.79 (0.05 - 1681.30) | 1.79 (0.05 - 1681.30) | 1.65 (0.35 - 1399.07) |  |
| **Log_e_ PMMoV (copies/ml)/Flow (MGD)** |  |  |  | *0.260* |
| N | 529 | 514 | 15 |  |
| Mean ± SE | 137.43 ± 20.32 | 133.52 ± 20.31 | 271.38 ± 172.92 |  |
| Median (min - max) | 3.01 (0.08 - 2482.84) | 3.01 (0.08 - 2482.84) | 3.38 (0.51 - 2131.11) |  |
| **Log_e_ CrAssphage (copies/ml)/Flow (MGD)** |  |  |  | *0.226* |
| N | 545 | 530 | 15 |  |
| Mean ± SE | 136.13 ± 19.77 | 132.10 ± 19.70 | 278.50 ± 178.56 |  |
| Median (min - max) | 2.94 (0.08 - 2372.31) | 2.93 (0.08 - 2372.31) | 3.61 (0.51 - 2251.95) |  |
| **Log_e_ RNase P (copies/ml)/**(**e^daily rainfall in inches^)** |  |  |  | *0.035* |
| N | 405 | 392 | 13 |  |
| Mean ± SE | 6.92 ± 0.09 | 6.88 ± 0.09 | 7.99 ± 0.49 |  |
| Median (min - max) | 6.86 (0.89 - 13.95) | 6.83 (0.89 - 13.95) | 7.75 (5.35 - 12.48) |  |
| **Log_e_ PMMoV (copies/ml)/**(**e^daily rainfall in inches^)** |  |  |  | *0.756* |
| N | 534 | 519 | 15 |  |
| Mean ± SE | 11.98 ± 0.11 | 11.97 ± 0.11 | 12.18 ± 0.67 |  |
| Median (min - max) | 12.42 (1.90 - 17.38) | 12.42 (1.90 - 17.38) | 12.53 (6.05 - 15.28) |  |
| **Log_e_ CrAssphage (copies/ml)/**(**e^daily rainfall in inches^)** |  |  |  | *0.665* |
| N | 549 | 534 | 15 |  |
| Mean ± SE | 11.97 ± 0.11 | 11.96 ± 0.11 | 12.24 ± 0.72 |  |
| Median (min - max) | 12.21 (1.86 - 19.03) | 12.20 (1.86 - 19.03) | 12.67 (5.98 - 15.76) |  |
| **Log_e_ RNase P (copies/ml)/(Flow*e^daily rainfall in inches^)** |  |  |  | *0.377* |
| N | 403 | 390 | 13 |  |
| Mean ± SE | 62.67 ± 11.64 | 60.79 ± 11.52 | 119.06 ± 107.07 |  |
| Median (min - max) | 1.73 (0.01 - 1681.30) | 1.74 (0.01 - 1681.30) | 1.54 (0.26 - 1399.07) |  |
| **Log_e_ PMMoV (copies/ml)/ (Flow*e^daily rainfall in inches^)** |  |  |  | *0.432* |
| N | 529 | 514 | 15 |  |
| Mean ± SE | 128.45 ± 19.35 | 125.85 ± 19.46 | 217.47 ± 148.46 |  |
| Median (min - max) | 2.90 (0.02 - 2482.84) | 2.89 (0.02 - 2482.84) | 3.38 (0.39 - 2131.11) |  |
| **Log_e_ CrAssphage (copies/ml)/ (Flow*e^daily rainfall in inches^)** |  |  |  | *0.378* |
| N | 545 | 530 | 15 |  |
| Mean ± SE | 126.94 ± 18.72 | 124.16 ± 18.76 | 225.14 ± 155.74 |  |
| Median (min - max) | 2.88 (0.02 - 2372.31) | 2.88 (0.02 - 2372.31) | 3.61 (0.37 - 2251.95) |  |

***Table C4: Comparison of the characteristics for Louisville/Jefferson County Metropolitan Sewer District (MSD) and Sanitation District No. 1 (SD1) of Northern Kentucky sites by combined sewer or non-combined sewer system sample location***

| **Variables** | **Total (N=650)** | **Combined sewer location (N=293)** | **Non-combined sewer location (N=357)** | **P Value** |
| --- | --- | --- | --- | --- |
| **Area** |  |  |  | *0.152* |
| N | 650 | 293 | 357 |  |
| Mean ± SE | 87.04 ± 3.86 | 93.15 ± 6.35 | 82.02 ± 4.71 |  |
| Median (min - max) | 54.85 (2.96 - 331.83) | 38.73 (2.96 - 279.73) | 54.85 (9.22 - 331.83) |  |
| **Population** |  |  |  | *<.001* |
| N | 650 | 293 | 357 |  |
| Mean ± SE | 88.08 ± 4.33 | 115.86 ± 7.91 | 65.28 ± 4.11 |  |
| Median (min - max) | 35.96 (4.90 - 349.85) | 39.19 (7.82 - 349.85) | 35.96 (4.90 - 295.91) |  |
| **Income** |  |  |  | *<.001* |
| N | 650 | 293 | 357 |  |
| Mean ± SE | 64.90 ± 0.92 | 56.94 ± 1.33 | 71.43 ± 1.17 |  |
| Median (min - max) | 59.01 (27.45 - 113.70) | 54.14 (27.45 - 103.30) | 63.64 (45.90 - 113.70) |  |
| **Flow (MGD)** |  |  |  | *<.001* |
| N | 561 | 265 | 296 |  |
| Mean ± SE | 16.20 ± 1.06 | 23.35 ± 1.94 | 9.79 ± 0.84 |  |
| Median (min - max) | 4.17 (0.01 - 169.35) | 2.00 (0.06 - 169.35) | 4.17 (0.01 - 115.61) |  |
| **Temperature (°F) at time of collection** |  |  |  | *0.671* |
| N | 497 | 198 | 299 |  |
| Mean ± SE | 38.41 ± 0.19 | 38.51 ± 0.30 | 38.35 ± 0.24 |  |
| Median (min - max) | 38.00 (33.00 - 77.00) | 38.00 (33.00 - 77.00) | 38.00 (33.00 - 69.00) |  |
| **Log_e_ RNase P (copies/ml)** |  |  |  | *0.846* |
| N | 463 | 211 | 252 |  |
| Mean ± SE | 7.26 ± 0.07 | 7.28 ± 0.10 | 7.25 ± 0.10 |  |
| Median (min - max) | 7.16 (3.92 - 13.95) | 7.19 (4.26 - 11.96) | 7.13 (3.92 - 13.95) |  |
| **Log_e_ PMMoV (copies/ml)** |  |  |  | *<.001* |
| N | 604 | 271 | 333 |  |
| Mean ± SE | 12.85 ± 0.06 | 12.64 ± 0.09 | 13.03 ± 0.07 |  |
| Median (min - max) | 12.87 (8.89 - 17.38) | 12.61 (8.89 - 16.87) | 12.99 (9.20 - 17.38) |  |
| **Log_e_ CrAssphage (copies/ml)** |  |  |  | *0.051* |
| N | 631 | 284 | 347 |  |
| Mean ± SE | 12.92 ± 0.06 | 12.79 ± 0.10 | 13.04 ± 0.08 |  |
| Median (min - max) | 12.60 (9.18 - 19.03) | 12.48 (9.40 - 19.03) | 12.74 (9.18 - 18.61) |  |
| **Log_e_ RNase P (copies/ml)/Flow (MGD)** |  |  |  | *<.001* |
| N | 403 | 190 | 213 |  |
| Mean ± SE | 65.74 ± 11.95 | 18.69 ± 2.65 | 107.72 ± 22.12 |  |
| Median (min - max) | 1.79 (0.05 - 1681.30) | 3.16 (0.06 - 157.81) | 1.67 (0.05 - 1681.30) |  |
| **Log_e_ PMMoV (copies/ml)/Flow (MGD)** |  |  |  | *<.001* |
| N | 529 | 247 | 282 |  |
| Mean ± SE | 137.43 ± 20.32 | 34.85 ± 4.28 | 227.28 ± 37.14 |  |
| Median (min - max) | 3.01 (0.08 - 2482.84) | 5.18 (0.08 - 259.89) | 3.00 (0.10 - 2482.84) |  |
| **Log_e_ CrAssphage (copies/ml)/Flow (MGD)** |  |  |  | *<.001* |
| N | 545 | 256 | 289 |  |
| Mean ± SE | 136.13 ± 19.77 | 35.02 ± 4.16 | 225.70 ± 36.31 |  |
| Median (min - max) | 2.94 (0.08 - 2372.31) | 5.22 (0.08 - 270.39) | 2.91 (0.10 - 2372.31) |  |
| **Log_e_ RNase P (copies/ml)/**(**e^daily rainfall in inches^)** |  |  |  | *0.575* |
| N | 463 | 211 | 252 |  |
| Mean ± SE | 6.88 ± 0.08 | 6.93 ± 0.11 | 6.84 ± 0.12 |  |
| Median (min - max) | 6.73 (0.89 - 13.95) | 6.74 (3.25 - 11.96) | 6.72 (0.89 - 13.95) |  |
| **Log_e_ PMMoV (copies/ml)/**(**e^daily rainfall in inches^)** |  |  |  | *0.122* |
| N | 604 | 271 | 333 |  |
| Mean ± SE | 12.15 ± 0.10 | 11.98 ± 0.14 | 12.29 ± 0.14 |  |
| Median (min - max) | 12.58 (1.90 - 17.38) | 12.33 (2.14 - 16.87) | 12.71 (1.90 - 17.38) |  |
| **Log_e_ CrAssphage (copies/ml)/**(**e^daily rainfall in inches^)** |  |  |  | *0.448* |
| N | 631 | 284 | 347 |  |
| Mean ± SE | 12.25 ± 0.10 | 12.16 ± 0.14 | 12.31 ± 0.14 |  |
| Median (min - max) | 12.35 (1.86 - 19.03) | 12.27 (1.86 - 19.03) | 12.37 (2.11 - 18.61) |  |
| **Log_e_ RNase P (copies/ml)/(Flow*e^daily rainfall in inches^)** |  |  |  | *<.001* |
| N | 403 | 190 | 213 |  |
| Mean ± SE | 62.67 ± 11.64 | 17.80 ± 2.56 | 102.70 ± 21.56 |  |
| Median (min - max) | 1.73 (0.01 - 1681.30) | 2.97 (0.06 - 157.81) | 1.58 (0.01 - 1681.30) |  |
| **Log_e_ PMMoV (copies/ml)/(Flow*e^daily rainfall in inches^)** |  |  |  | *<.001* |
| N | 529 | 247 | 282 |  |
| Mean ± SE | 128.45 ± 19.35 | 32.88 ± 4.09 | 212.15 ± 35.41 |  |
| Median (min - max) | 2.90 (0.02 - 2482.84) | 4.34 (0.02 - 259.89) | 2.82 (0.02 - 2482.84) |  |
| **Log_e_ CrAssphage (copies/ml)/(Flow*e^daily rainfall in inches^)** |  |  |  | *<.001* |
| N | 545 | 256 | 289 |  |
| Mean ± SE | 126.94 ± 18.72 | 33.15 ± 3.99 | 210.01 ± 34.43 |  |
| Median (min - max) | 2.88 (0.02 - 2372.31) | 4.12 (0.02 - 270.39) | 2.83 (0.02 - 2372.31) |  |

***Table C5: Comparison of the characteristics for Louisville/Jefferson County Metropolitan Sewer District (MSD) and Sanitation District No. 1 (SD1) of Northern Kentucky sites by sample collection types: street line manhole, pump station, and water quality treatment center***

| **Variables** | **Total (N=650)** | **Manhole (N=299)** | **Pump Station (N=120)** | **Treatment Center (N=231)** | **P Value** |
| --- | --- | --- | --- | --- | --- |
| **Area** |  |  |  |  | *<.001* |
| N | 650 | 299 | 120 | 231 |  |
| Mean ± SE | 87.04 ± 3.86 | 46.81 ± 2.26 | 10.84 ± 0.80 | 178.69 ± 7.14 |  |
| Median (min - max) | 54.85 (2.96 - 331.83) | 36.97 (2.96 - 183.81) | 5.07 (4.88 - 36.99) | 124.34 (30.96 - 331.83) |  |
| **Population** |  |  |  |  | *<.001* |
| N | 650 | 299 | 120 | 231 |  |
| Mean ± SE | 88.08 ± 4.33 | 54.11 ± 2.37 | 11.44 ± 0.48 | 171.86 ± 9.45 |  |
| Median (min - max) | 35.96 (4.90 - 349.85) | 35.96 (8.07 - 139.25) | 10.74 (7.82 - 31.14) | 90.21 (4.90 - 349.85) |  |
| **Income** |  |  |  |  | *<.001* |
| N | 650 | 299 | 120 | 231 |  |
| Mean ± SE | 64.90 ± 0.92 | 60.21 ± 0.70 | 56.43 ± 3.13 | 75.37 ± 1.60 |  |
| Median (min - max) | 59.01 (27.45 - 113.70) | 61.84 (41.75 - 98.43) | 27.69 (27.45 - 103.30) | 71.90 (48.74 - 113.70) |  |
| **Flow (MGD)** |  |  |  |  | *<.001* |
| N | 561 | 264 | 99 | 198 |  |
| Mean ± SE | 16.20 ± 1.06 | 5.31 ± 0.44 | 13.67 ± 1.87 | 31.98 ± 2.39 |  |
| Median (min - max) | 4.17 (0.01 - 169.35) | 3.08 (0.01 - 23.06) | 0.98 (0.25 - 39.78) | 6.56 (0.02 - 169.35) |  |
| **Temperature (°F) at time of collection** |  |  |  |  | *<.001* |
| N | 497 | 263 | 99 | 135 |  |
| Mean ± SE | 38.41 ± 0.19 | 39.14 ± 0.31 | 37.48 ± 0.29 | 37.68 ± 0.20 |  |
| Median (min - max) | 38.00 (33.00 - 77.00) | 38.00 (33.00 - 77.00) | 37.00 (33.00 - 48.00) | 37.00 (34.00 - 46.00) |  |
| **Log_e_ RNase P (copies/ml)** |  |  |  |  | *0.003* |
| N | 463 | 234 | 94 | 135 |  |
| Mean ± SE | 7.26 ± 0.07 | 7.39 ± 0.10 | 7.48 ± 0.16 | 6.89 ± 0.12 |  |
| Median (min - max) | 7.16 (3.92 - 13.95) | 7.35 (3.92 - 13.95) | 7.44 (4.26 - 11.96) | 6.73 (4.27 - 11.74) |  |
| **Log_e_ PMMoV (copies/ml)** |  |  |  |  | *0.255* |
| N | 604 | 277 | 111 | 216 |  |
| Mean ± SE | 12.85 ± 0.06 | 12.87 ± 0.09 | 13.02 ± 0.13 | 12.75 ± 0.09 |  |
| Median (min - max) | 12.87 (8.89 - 17.38) | 12.86 (9.20 - 17.38) | 13.02 (10.06 - 16.62) | 12.80 (8.89 - 17.29) |  |
| **Log_e_ CrAssphage (copies/ml)** |  |  |  |  | *<.001* |
| N | 631 | 289 | 115 | 227 |  |
| Mean ± SE | 12.92 ± 0.06 | 12.95 ± 0.09 | 13.36 ± 0.15 | 12.67 ± 0.11 |  |
| Median (min - max) | 12.60 (9.18 - 19.03) | 12.67 (9.71 - 18.61) | 13.17 (9.75 - 19.03) | 12.36 (9.18 - 18.65) |  |
| **Log_e_ RNase P (copies/ml)/Flow (MGD)** |  |  |  |  | *<.001* |
| N | 403 | 207 | 79 | 117 |  |
| Mean ± SE | 65.74 ± 11.95 | 120.13 ± 22.46 | 11.38 ± 1.31 | 6.23 ± 5.09 |  |
| Median (min - max) | 1.79 (0.05 - 1681.30) | 1.99 (0.23 - 1681.30) | 8.11 (0.14 - 35.88) | 1.33 (0.05 - 597.02) |  |
| **Log_e_ PMMoV (copies/ml)/Flow (MGD)** |  |  |  |  | *<.001* |
| N | 529 | 247 | 93 | 189 |  |
| Mean ± SE | 137.43 ± 20.32 | 281.10 ± 41.57 | 22.33 ± 2.30 | 6.32 ± 4.31 |  |
| Median (min - max) | 3.01 (0.08 - 2482.84) | 4.75 (0.49 - 2482.84) | 13.02 (0.28 - 65.42) | 1.62 (0.08 - 816.28) |  |
| **Log_e_ CrAssphage (copies/ml)/Flow (MGD)** |  |  |  |  | *<.001* |
| N | 545 | 255 | 95 | 195 |  |
| Mean ± SE | 136.13 ± 19.77 | 277.75 ± 40.35 | 22.58 ± 2.38 | 6.25 ± 4.30 |  |
| Median (min - max) | 2.94 (0.08 - 2372.31) | 4.90 (0.45 - 2372.31) | 13.25 (0.26 - 75.93) | 1.81 (0.08 - 839.12) |  |
| **Log_e_ RNase P (copies/ml)/**(**e^daily rainfall in inches^)** |  |  |  |  | *0.092* |
| N | 463 | 234 | 94 | 135 |  |
| Mean ± SE | 6.88 ± 0.08 | 6.97 ± 0.12 | 7.06 ± 0.17 | 6.60 ± 0.15 |  |
| Median (min - max) | 6.73 (0.89 - 13.95) | 6.83 (1.27 - 13.95) | 6.87 (3.25 - 11.96) | 6.56 (0.89 - 11.74) |  |
| **Log_e_ PMMoV (copies/ml)/**(**e^daily rainfall in inches^)** |  |  |  |  | *0.448* |
| N | 604 | 277 | 111 | 216 |  |
| Mean ± SE | 12.15 ± 0.10 | 12.12 ± 0.15 | 12.41 ± 0.20 | 12.06 ± 0.17 |  |
| Median (min - max) | 12.58 (1.90 - 17.38) | 12.53 (2.64 - 17.38) | 12.71 (6.58 - 16.62) | 12.50 (1.90 - 17.29) |  |
| **Log_e_ CrAssphage (copies/ml)/**(**e^daily rainfall in inches^)** |  |  |  |  | *0.034* |
| N | 631 | 289 | 115 | 227 |  |
| Mean ± SE | 12.25 ± 0.10 | 12.21 ± 0.15 | 12.77 ± 0.22 | 12.02 ± 0.17 |  |
| Median (min - max) | 12.35 (1.86 - 19.03) | 12.35 (2.77 - 18.61) | 12.94 (6.82 - 19.03) | 12.15 (1.86 - 18.65) |  |
| **Log_e_ RNase P (copies/ml)/(Flow*e^daily rainfall in inches^)** |  |  |  |  | *<.001* |
| N | 403 | 207 | 79 | 117 |  |
| Mean ± SE | 62.67 ± 11.64 | 114.46 ± 21.90 | 10.63 ± 1.25 | 6.19 ± 5.09 |  |
| Median (min - max) | 1.73 (0.01 - 1681.30) | 1.89 (0.06 - 1681.30) | 7.23 (0.11 - 35.88) | 1.22 (0.01 - 597.02) |  |
| **Log_e_ PMMoV (copies/ml)/(Flow*e^daily rainfall in inches^)** |  |  |  |  | *<.001* |
| N | 529 | 247 | 93 | 189 |  |
| Mean ± SE | 128.45 ± 19.35 | 262.38 ± 39.66 | 21.12 ± 2.22 | 6.22 ± 4.31 |  |
| Median (min - max) | 2.90 (0.02 - 2482.84) | 3.36 (0.12 - 2482.84) | 12.86 (0.18 - 65.42) | 0.97 (0.02 - 816.28) |  |
| **Log_e_ CrAssphage (copies/ml)/(Flow*e^daily rainfall in inches^)** |  |  |  |  | *<.001* |
| N | 545 | 255 | 95 | 195 |  |
| Mean ± SE | 126.94 ± 18.72 | 258.63 ± 38.27 | 21.37 ± 2.30 | 6.15 ± 4.30 |  |
| Median (min - max) | 2.88 (0.02 - 2372.31) | 3.34 (0.13 - 2372.31) | 12.50 (0.17 - 75.93) | 1.04 (0.02 - 839.12) |  |

***Table C6: Comparison of the characteristics for nested contributing sites leading to*** ***Morris Forman Water Quality Treatment Center (MFWQTC) compared to those of MFWQTC***

| **Variables** | **Total (N=265)** | **Leading to MFWQTC  (N=198)** | **MFWQTC (N=67)** | **P Value** |
| --- | --- | --- | --- | --- |
| **Area** |  |  |  | *<.001* |
| N | 265 | 198 | 67 |  |
| Mean ± SE | 97.75 ± 6.91 | 36.17 ± 3.08 | 279.73 ± 0.00 |  |
| Median (min - max) | 80.13 (2.96 - 279.73) | 8.76 (2.96 - 111.56) | 279.73 (279.73 - 279.73) |  |
| **Population** |  |  |  | *<.001* |
| N | 265 | 198 | 67 |  |
| Mean ± SE | 122.84 ± 8.60 | 46.02 ± 3.78 | 349.85 ± 0.00 |  |
| Median (min - max) | 99.06 (7.82 - 349.85) | 10.97 (7.82 - 139.25) | 349.85 (349.85 - 349.85) |  |
| **Population density** |  |  |  | *<.001* |
| N | 265 | 198 | 67 |  |
| Mean ± SE | 1541.10 ± 34.42 | 1639.38 ± 43.94 | 1250.67 ± 0.00 |  |
| Median (min - max) | 1250.67 (900.35 - 2730.46) | 1425.66 (900.35 - 2730.46) | 1250.67 (1250.67 - 1250.67) |  |
| **Income** |  |  |  | *0.154* |
| N | 265 | 198 | 67 |  |
| Mean ± SE | 57.72 ± 1.46 | 58.93 ± 1.95 | 54.14 ± 0.00 |  |
| Median (min - max) | 54.14 (27.45 - 103.30) | 58.65 (27.45 - 103.30) | 54.14 (54.14 - 54.14) |  |
| **Flow (MGD)** |  |  |  | *<.001* |
| N | 265 | 198 | 67 |  |
| Mean ± SE | 23.35 ± 1.94 | 8.02 ± 1.02 | 68.68 ± 2.98 |  |
| Median (min - max) | 2.00 (0.06 - 169.35) | 1.49 (0.06 - 39.78) | 62.99 (1.24 - 169.35) |  |
| **Temperature (°F) at time of collection** |  |  |  |  |
| N | 198 | 198 | 0 |  |
| Mean ± SE | 38.51 ± 0.30 | 38.51 ± 0.30 |  |  |
| Median (min - max) | 38.00 (33.00 - 77.00) | 38.00 (33.00 - 77.00) |  |  |
| **Log_e_ RNase P (copies/ml)** |  |  |  | *<.001* |
| N | 190 | 159 | 31 |  |
| Mean ± SE | 7.31 ± 0.11 | 7.47 ± 0.11 | 6.50 ± 0.24 |  |
| Median (min - max) | 7.36 (4.26 - 11.96) | 7.46 (4.26 - 11.96) | 6.30 (4.27 - 10.00) |  |
| **Log_e_ PMMoV (copies/ml)** |  |  |  | *0.035* |
| N | 247 | 182 | 65 |  |
| Mean ± SE | 12.55 ± 0.10 | 12.67 ± 0.11 | 12.21 ± 0.18 |  |
| Median (min - max) | 12.53 (8.89 - 16.87) | 12.56 (9.26 - 16.87) | 12.33 (8.89 - 15.25) |  |
| **Log_e_ CrAssphage (copies/ml)** |  |  |  | *0.023* |
| N | 256 | 189 | 67 |  |
| Mean ± SE | 12.65 ± 0.10 | 12.79 ± 0.11 | 12.27 ± 0.22 |  |
| Median (min - max) | 12.38 (9.40 - 19.03) | 12.56 (9.71 - 19.03) | 12.05 (9.40 - 18.65) |  |
| **Log_e_ RNase P (copies/ml)/Flow (MGD)** |  |  |  | *0.002* |
| N | 190 | 159 | 31 |  |
| Mean ± SE | 18.69 ± 2.65 | 22.31 ± 3.08 | 0.11 ± 0.01 |  |
| Median (min - max) | 3.16 (0.06 - 157.81) | 4.28 (0.14 - 157.81) | 0.10 (0.06 - 0.17) |  |
| **Log_e_ PMMoV (copies/ml)/Flow (MGD)** |  |  |  | *<.001* |
| N | 247 | 182 | 65 |  |
| Mean ± SE | 34.85 ± 4.28 | 47.14 ± 5.53 | 0.44 ± 0.18 |  |
| Median (min - max) | 5.18 (0.08 - 259.89) | 11.15 (0.28 - 259.89) | 0.19 (0.08 - 8.96) |  |
| **Log_e_ CrAssphage (copies/ml)/Flow (MGD)** |  |  |  | *<.001* |
| N | 256 | 189 | 67 |  |
| Mean ± SE | 35.02 ± 4.16 | 47.28 ± 5.36 | 0.43 ± 0.18 |  |
| Median (min - max) | 5.22 (0.08 - 270.39) | 11.27 (0.26 - 270.39) | 0.19 (0.08 - 9.23) |  |
| **Log_e_ RNase P (copies/ml)/**(**e^daily rainfall in inches^)** |  |  |  | *0.035* |
| N | 190 | 159 | 31 |  |
| Mean ± SE | 6.93 ± 0.12 | 7.04 ± 0.13 | 6.36 ± 0.27 |  |
| Median (min - max) | 6.78 (3.25 - 11.96) | 6.88 (3.25 - 11.96) | 6.30 (3.80 - 10.00) |  |
| **Log_e_ PMMoV (copies/ml)/**(**e^daily rainfall in inches^)** |  |  |  | *0.168* |
| N | 247 | 182 | 65 |  |
| Mean ± SE | 11.83 ± 0.15 | 11.95 ± 0.17 | 11.49 ± 0.30 |  |
| Median (min - max) | 12.16 (2.14 - 16.87) | 12.17 (5.37 - 16.87) | 12.12 (2.14 - 15.25) |  |
| **Log_e_ CrAssphage (copies/ml)/**(**e^daily rainfall in inches^)** |  |  |  | *0.133* |
| N | 256 | 189 | 67 |  |
| Mean ± SE | 11.96 ± 0.15 | 12.09 ± 0.17 | 11.58 ± 0.32 |  |
| Median (min - max) | 12.17 (1.86 - 19.03) | 12.31 (5.83 - 19.03) | 11.81 (1.86 - 18.65) |  |
| **Log_e_ RNase P (copies/ml)/(Flow*e^daily rainfall in inches^)** |  |  |  | *0.002* |
| N | 190 | 159 | 31 |  |
| Mean ± SE | 17.80 ± 2.56 | 21.25 ± 2.98 | 0.10 ± 0.01 |  |
| Median (min - max) | 2.97 (0.06 - 157.81) | 4.28 (0.11 - 157.81) | 0.10 (0.06 - 0.17) |  |
| **Log_e_ PMMoV (copies/ml)/(Flow*e^daily rainfall in inches^)** |  |  |  | *<.001* |
| N | 247 | 182 | 65 |  |
| Mean ± SE | 32.88 ± 4.09 | 44.46 ± 5.29 | 0.43 ± 0.18 |  |
| Median (min - max) | 4.34 (0.02 - 259.89) | 9.54 (0.18 - 259.89) | 0.18 (0.02 - 8.96) |  |
| **Log_e_ CrAssphage (copies/ml)/(Flow*e^daily rainfall in inches^)** |  |  |  | *<.001* |
| N | 256 | 189 | 67 |  |
| Mean ± SE | 33.15 ± 3.99 | 44.75 ± 5.15 | 0.43 ± 0.18 |  |
| Median (min - max) | 4.12 (0.02 - 270.39) | 7.59 (0.17 - 270.39) | 0.19 (0.02 - 9.23) |  |

***Table C7: Comparison of the characteristics for nested contributing sites leading to Derek R. Guthrie Water Quality Treatment Center (DRGWQTC) compared to those of DRGWQTC***

| **Variables** | **Total (N=199)** | **Leading to DRGWQTC (N=165)** | **DRGWQTC (N=34)** | **P Value** |
| --- | --- | --- | --- | --- |
| **Area** |  |  |  | *<.001* |
| N | 199 | 165 | 34 |  |
| Mean ± SE | 83.77 ± 8.04 | 32.66 ± 0.97 | 331.83 ± 0.00 |  |
| Median (min - max) | 36.97 (20.61 - 331.83) | 27.52 (20.61 - 54.85) | 331.83 (331.83 - 331.83) |  |
| **Population** |  |  |  | *<.001* |
| N | 199 | 165 | 34 |  |
| Mean ± SE | 84.35 ± 6.93 | 40.76 ± 1.45 | 295.91 ± 0.00 |  |
| Median (min - max) | 46.66 (22.44 - 295.91) | 35.96 (22.44 - 73.67) | 295.91 (295.91 - 295.91) |  |
| **Population density** |  |  |  | *<.001* |
| N | 199 | 165 | 34 |  |
| Mean ± SE | 1162.23 ± 12.35 | 1217.97 ± 10.54 | 891.74 ± 0.00 |  |
| Median (min - max) | 1216.28 (891.74 - 1343.04) | 1262.22 (961.67 - 1343.04) | 891.74 (891.74 - 891.74) |  |
| **Income** |  |  |  | *0.128* |
| N | 199 | 165 | 34 |  |
| Mean ± SE | 55.02 ± 0.43 | 55.31 ± 0.51 | 53.58 ± 0.00 |  |
| Median (min - max) | 53.58 (45.90 - 63.64) | 53.54 (45.90 - 63.64) | 53.58 (53.58 - 53.58) |  |
| **Flow (MGD)** |  |  |  | *<.001* |
| N | 194 | 165 | 29 |  |
| Mean ± SE | 12.81 ± 1.22 | 7.08 ± 0.65 | 45.39 ± 3.16 |  |
| Median (min - max) | 4.17 (0.01 - 115.61) | 4.17 (0.01 - 23.06) | 40.74 (30.55 - 115.61) |  |
| **Temperature (°F) at time of collection** |  |  |  | *0.012* |
| N | 198 | 164 | 34 |  |
| Mean ± SE | 38.52 ± 0.34 | 38.90 ± 0.39 | 36.68 ± 0.34 |  |
| Median (min - max) | 38.00 (33.00 - 69.00) | 38.00 (33.00 - 69.00) | 37.00 (34.00 - 42.00) |  |
| **Log_e_ RNase P (copies/ml)** |  |  |  | *0.106* |
| N | 148 | 127 | 21 |  |
| Mean ± SE | 7.50 ± 0.14 | 7.58 ± 0.15 | 6.96 ± 0.28 |  |
| Median (min - max) | 7.58 (3.92 - 13.95) | 7.62 (3.92 - 13.95) | 6.85 (4.79 - 8.86) |  |
| **Log_e_ PMMoV (copies/ml)** |  |  |  | *0.919* |
| N | 191 | 158 | 33 |  |
| Mean ± SE | 13.01 ± 0.10 | 13.00 ± 0.12 | 13.03 ± 0.20 |  |
| Median (min - max) | 12.95 (9.20 - 17.38) | 12.87 (9.20 - 17.38) | 13.00 (10.19 - 15.08) |  |
| **Log_e_ CrAssphage (copies/ml)** |  |  |  | *0.363* |
| N | 194 | 161 | 33 |  |
| Mean ± SE | 12.90 ± 0.10 | 12.94 ± 0.11 | 12.70 ± 0.25 |  |
| Median (min - max) | 12.66 (9.18 - 16.61) | 12.69 (10.03 - 16.61) | 12.53 (9.18 - 15.84) |  |
| **Log_e_ RNase P (copies/ml)/Flow (MGD)** |  |  |  | *0.061* |
| N | 146 | 127 | 19 |  |
| Mean ± SE | 152.20 ± 31.36 | 174.94 ± 35.63 | 0.16 ± 0.01 |  |
| Median (min - max) | 1.36 (0.05 - 1681.30) | 1.62 (0.23 - 1681.30) | 0.16 (0.05 - 0.24) |  |
| **Log_e_ PMMoV (copies/ml)/Flow (MGD)** |  |  |  | *0.009* |
| N | 186 | 158 | 28 |  |
| Mean ± SE | 338.37 ± 54.42 | 398.28 ± 62.89 | 0.30 ± 0.01 |  |
| Median (min - max) | 2.57 (0.10 - 2482.84) | 3.00 (0.49 - 2482.84) | 0.32 (0.10 - 0.42) |  |
| **Log_e_ CrAssphage (copies/ml)/Flow (MGD)** |  |  |  | *0.007* |
| N | 190 | 161 | 29 |  |
| Mean ± SE | 337.08 ± 53.34 | 397.75 ± 61.78 | 0.30 ± 0.01 |  |
| Median (min - max) | 2.55 (0.10 - 2372.31) | 2.90 (0.45 - 2372.31) | 0.30 (0.10 - 0.42) |  |
| **Log_e_ RNase P (copies/ml)/**(**e^daily rainfall in inches^)** |  |  |  | *0.103* |
| N | 148 | 127 | 21 |  |
| Mean ± SE | 6.93 ± 0.18 | 7.05 ± 0.19 | 6.22 ± 0.39 |  |
| Median (min - max) | 7.08 (1.27 - 13.95) | 7.30 (1.27 - 13.95) | 5.81 (1.40 - 8.86) |  |
| **Log_e_ PMMoV (copies/ml)/**(**e^daily rainfall in inches^)** |  |  |  | *0.917* |
| N | 191 | 158 | 33 |  |
| Mean ± SE | 12.09 ± 0.20 | 12.09 ± 0.22 | 12.14 ± 0.44 |  |
| Median (min - max) | 12.53 (2.61 - 17.38) | 12.54 (2.64 - 17.38) | 12.49 (2.61 - 15.08) |  |
| **Log_e_ CrAssphage (copies/ml)/**(**e^daily rainfall in inches^)** |  |  |  | *0.684* |
| N | 194 | 161 | 33 |  |
| Mean ± SE | 11.98 ± 0.19 | 12.01 ± 0.21 | 11.81 ± 0.45 |  |
| Median (min - max) | 12.23 (2.68 - 16.61) | 12.29 (2.77 - 16.61) | 12.12 (2.68 - 15.84) |  |
| **Log_e_ RNase P (copies/ml)/(Flow*e^daily rainfall in inches^)** |  |  |  | *0.067* |
| N | 146 | 127 | 19 |  |
| Mean ± SE | 144.91 ± 30.59 | 166.57 ± 34.78 | 0.14 ± 0.01 |  |
| Median (min - max) | 1.21 (0.01 - 1681.30) | 1.47 (0.06 - 1681.30) | 0.14 (0.01 - 0.24) |  |
| **Log_e_ PMMoV (copies/ml)/(Flow*e^daily rainfall in inches^)** |  |  |  | *0.010* |
| N | 186 | 158 | 28 |  |
| Mean ± SE | 315.53 ± 51.95 | 371.40 ± 60.10 | 0.29 ± 0.02 |  |
| Median (min - max) | 2.15 (0.02 - 2482.84) | 2.88 (0.12 - 2482.84) | 0.32 (0.02 - 0.41) |  |
| **Log_e_ CrAssphage (copies/ml)/(Flow*e^daily rainfall in inches^)** |  |  |  | *0.008* |
| N | 190 | 161 | 29 |  |
| Mean ± SE | 313.32 ± 50.63 | 369.71 ± 58.67 | 0.28 ± 0.02 |  |
| Median (min - max) | 2.01 (0.02 - 2372.31) | 2.83 (0.13 - 2372.31) | 0.30 (0.02 - 0.42) |  |

***Table C8: Regression analysis of the samples of Louisville/Jefferson County Metropolitan Sewer District (MSD) sites***

| **Univariable Regression** | | | | | | | | | |
| --- | --- | --- | --- | --- | --- | --- | --- | --- | --- |
| **Variables** | **Log_e_** **RNase P**  **(copies/ml)** | | | **Log_e_ PMMoV**  **(copies/ml)** | | | **Log_e_ CrAssphage (copies/ml)** | | |
|  | **Estimate** | **SE** | **P Value** | **Estimate** | **SE** | **P Value** | **Estimate** | **SE** | **P Value** |
| **Income** | 0.000 | 0.003 | 0.9308 | -0.002 | 0.003 | 0.3611 | -0.005 | 0.003 | 0.0443 |
| **Population** | -0.003 | 0.001 | 0.0005 | -0.001 | 0.001 | 0.0074 | -0.002 | 0.001 | 0.0044 |
| **Area** | -0.003 | 0.001 | 0.0002 | -0.001 | 0.001 | 0.033 | -0.002 | 0.001 | 0.0049 |
| **Population density** | 0.000 | 0.000 | 0.3187 | 0.000 | 0.000 | 0.3722 | 0.000 | 0.000 | 0.8233 |
| **Composite or grab** | -1.158 | 0.428 | 0.0071 | -0.424 | 0.374 | 0.2583 | -0.504 | 0.388 | 0.1953 |
| **Combined sewer or Non-combined sewer** | -0.074 | 0.153 | 0.6265 | -0.419 | 0.123 | 0.0007 | -0.179 | 0.127 | 0.1597 |
| **Manhole** | 0.261 | 0.152 | 0.0866 | 0.031 | 0.124 | 0.8012 | -0.025 | 0.127 | 0.8415 |
| **Treatment center** | -0.580 | 0.165 | 0.0005 | -0.141 | 0.129 | 0.2736 | -0.304 | 0.131 | 0.0211 |
| **Pump station** | -0.580 | 0.165 | 0.0005 | -0.141 | 0.129 | 0.2736 | -0.304 | 0.131 | 0.0211 |
| **Temperature (°F) at time of collection** | -0.017 | 0.018 | 0.363 | 0.014 | 0.016 | 0.3834 | -0.001 | 0.016 | 0.9592 |
| **Rainfall (inches)** | -0.248 | 0.135 | 0.0668 | -0.242 | 0.112 | 0.0319 | -0.094 | 0.117 | 0.4242 |
| **Flow (MGD)** | -0.007 | 0.004 | 0.0555 | -0.005 | 0.002 | 0.0557 | -0.003 | 0.003 | 0.1788 |
| **Multivariable Regression** | | | | | | | | | |
| **Variables** | **Log_e_ RNase P**  **(copies/ml)** | | | **Log_e_ PMMoV**  **(copies/ml)** | | | **Log_e_ CrAssphage (copies/ml)** | | |
|  | **Estimate** | **SE** | **P Value** | **Estimate** | **SE** | **P Value** | **Estimate** | **SE** | **P Value** |
| **Income** | **NI^a^** |  |  | **NI** |  |  | -0.008 | 0.003 | 0.0112 |
| **Population** | -0.003 | 0.003 | 0.389 | -0.001 | 0.002 | 0.589 | -0.004 | 0.003 | 0.1355 |
| **Area** | 0.002 | 0.004 | 0.6945 | 0.000 | 0.003 | 0.8979 | 0.002 | 0.003 | 0.5027 |
| **Composite or grab** | -1.077 | 0.422 | 0.0112 |  |  |  | **NI** |  |  |
| **Combined sewer or Non-combined sewer** | **NI** |  |  | -0.372 | 0.152 | 0.015 | **NI** |  |  |
| **Treatment center** | -0.438 | 0.284 | 0.1242 | **NI** |  |  | 0.061 | 0.249 | 0.8049 |
| **Rainfall (inches)** | **NI** |  |  | -0.263 | 0.111 | 0.0185 | **NI** |  |  |
| **Pump station** | 0.000 | . | . | **NI** |  |  | 0.000 | . |  |

^a^: NI= not included in model.

***Table C9: Regression analysis of the samples of Morris Forman Water Quality Treatment Center (MFWQTC) compared to those of nested contributing sites leading to MFWQTC***

| **Univariable Regression** | | | | | | | | | |
| --- | --- | --- | --- | --- | --- | --- | --- | --- | --- |
| **Variables** | **Log_e_ RNase P (copies/ml)** | | | **Log_e_ PMMoV (copies/ml)** | | | **Log_e_ CrAssphage (copies/ml)** | | |
|  | **Estimate** | **SE** | **P Value** | **Estimate** | **SE** | **P Value** | **Estimate** | **SE** | **P Value** |
| **Income** | 0.007 | 0.004 | 0.0961 | -0.001 | 0.004 | 0.7863 | -0.003 | 0.004 | 0.485 |
| **Population** | -0.003 | 0.001 | 0.0004 | -0.002 | 0.001 | 0.0146 | -0.002 | 0.001 | 0.0119 |
| **Area** | -0.004 | 0.001 | 0.0004 | -0.002 | 0.001 | 0.0148 | -0.002 | 0.001 | 0.0124 |
| **Population density** | 0.000 | 0.000 | 0.7021 | 0.000 | 0.000 | 0.8804 | 0.000 | 0.000 | 0.4589 |
| **Composite or grab** | -0.283 | 0.745 | 0.7048 | -0.382 | 0.680 | 0.5749 | -0.045 | 0.726 | 0.9504 |
| **Manhole** | -0.004 | 0.217 | 0.9864 | -0.216 | 0.199 | 0.278 | -0.426 | 0.207 | 0.0401 |
| **Treatment center** | -0.976 | 0.281 | 0.0006 | -0.457 | 0.216 | 0.0348 | -0.519 | 0.226 | 0.0225 |
| **Pump station** | -0.976 | 0.281 | 0.0006 | -0.457 | 0.216 | 0.0348 | -0.519 | 0.226 | 0.0225 |
| **Temperature (°F) at time of collection** | -0.018 | 0.028 | 0.5087 | 0.004 | 0.026 | 0.864 | 0.001 | 0.025 | 0.96 |
| **Rainfall (inches)** | 0.181 | 0.584 | 0.7565 | -0.429 | 0.259 | 0.0988 | -0.651 | 0.274 | 0.0184 |
| **Flow (MGD)** | -0.010 | 0.004 | 0.0263 | -0.002 | 0.003 | 0.4969 | -0.002 | 0.003 | 0.55 |
| **Multivariable Regression** | | | | | | | | | |
| **Variables** | **Log_e_ RNase P (copies/ml)** | | | **Log_e_ PMMoV (copies/ml)** | | | **Log_e_ CrAssphage (copies/ml)** | | |
|  | **Estimate** | **SE** | **P Value** | **Estimate** | **SE** | **P Value** | **Estimate** | **SE** | **P Value** |
| **Population** | -0.081 | 0.042 | 0.0542 | -0.020 | 0.038 | 0.5952 | 0.039 | 0.043 | 0.3666 |
| **Area** | 0.097 | 0.051 | 0.0596 | 0.021 | 0.046 | 0.649 | -0.041 | 0.052 | 0.4286 |
| **Composite or grab** | **NI^a^** |  |  | 0.514 | 0.701 | 0.4643 | **NI** |  |  |
| **Manhole** | **NI** |  |  | **NI** |  |  | -1.261 | 0.332 | 0.0002 |
| **Treatment center** | -0.981 | 0.897 | 0.2752 | **NI** |  |  | -2.997 | 1.155 | 0.01 |
| **Pump station** | 0.000 | . | . | **NI** |  |  | 0.000 | . | . |
| **Rainfall (inches)** | **NI** |  |  | 0.000 | . | . | -0.606 | 0.266 | 0.0237 |
| **Flow (MGD)** | 0.014 | 0.009 | 0.0974 | **NI** |  |  | **NI** |  |  |

^a^: NI= not included in model.

***Table C9: Regression analysis of the samples of Derek R. Guthrie Water Quality Treatment Center (DRGWQTC) compared to those of nested contributing sites leading to DRGWQTC***

| **Univariable Regression** | | | | | | | | | | | | |
| --- | --- | --- | --- | --- | --- | --- | --- | --- | --- | --- | --- | --- |
| **Variables** | **Log_e_ RNase P** | | | | **Log_e_ PMMoV** | | | | **Log_e_ CrAssphage** | | | |
|  | **Estimate** | **SE** | **P Value** | **Estimate** | | **SE** | **P Value** | **Estimate** | | **SE** | **P Value** |  |
| **Income** | 0.014 | 0.022 | 0.5395 | -0. 073 | | 0.016 | <.0001 | -0.073 | | 0.016 | <.0001 |  |
| **Population** | -0.002 | 0.001 | 0.1307 | 0.000 | | 0.001 | 0.8977 | -0.001 | | 0.001 | 0.2897 |  |
| **Area** | -0.002 | 0.001 | 0.1192 | 0.000 | | 0.001 | 0.9498 | -0.001 | | 0.001 | 0.2861 |  |
| **Population density** | 0.001 | 0.001 | 0.3059 | 0.001 | | 0.001 | 0.0652 | 0.001 | | 0.001 | 0.0114 |  |
| **Composite or grab** | -1.840 | 0.621 | 0.0036 | -0.291 | | 0.514 | 0.5724 | -0.524 | | 0.498 | 0.2943 |  |
| **Manhole** | 0.628 | 0.386 | 0.1058 | -0.028 | | 0.273 | 0.9188 | 0.240 | | 0.264 | 0.3634 |  |
| **Treatment center** | -0.628 | 0.386 | 0.1058 | 0.028 | | 0.273 | 0.9188 | -0.240 | | 0.264 | 0.3634 |  |
| **Pump station** | -0.628 | 0.386 | 0.1058 | 0.028 | | 0.273 | 0.9188 | -0.240 | | 0.264 | 0.3634 |  |
| **Temperature (°F) at time of collection** | -0.021 | 0.026 | 0.4197 | 0.028 | | 0.024 | 0.2478 | 0.001 | | 0.024 | 0.9777 |  |
| **Rainfall (inches)** | -0.340 | 0.191 | 0.0764 | -0.340 | | 0.162 | 0.0367 | -0.055 | | 0.159 | 0.7293 |  |
| **Flow (MGD)** | -0.004 | 0.008 | 0.5759 | -0.007 | | 0.006 | 0.2395 | -0.009 | | 0.006 | 0.1164 |  |
| **Multivariable Regression** | | | | | | | | | | | | |
|  | **Log_e_ RNase P** | | | | **Log_e_ PMMoV** | | | | **Log_e_ CrAssphage** | | | |
| **Variables** | **Estimate** | **SE** | **P Value** | **Estimate** | | **SE** | **P Value** | **Estimate** | | **SE** | **P Value** |  |
| **Composite or grab** | -1.840 | 0.621 | 0.0036 | **NI^a^** | |  |  | **NI** | |  |  |  |
| **Income** | **NI** |  |  | -0.073 | | 0.016 | <.0001 | -0.067 | | 0.017 | <.0001 |  |
| **Population density** | **NI** |  |  | **NI** | |  |  | 0.001 | | 0.001 | 0.364 |  |
| **Rainfall (inches)** | **NI** |  |  | -0.339 | | 0.154 | 0.0289 | **NI** | |  |  |  |

^a^: NI= not included in model.
